# Exploring the Sustainable Exploitation of Bioactive Compounds in *Pelargonium* sp.: Beyond a Fragrant Plant

**DOI:** 10.3390/plants12244123

**Published:** 2023-12-10

**Authors:** Sara Roman, Catalina Voaides, Narcisa Babeanu

**Affiliations:** Faculty of Biotechnologies, University of Agronomic Sciences and Veterinary Medicine of Bucharest, 59 Marasti Blvd., 011464 Bucharest, Romania; sara.roman@usamv.ro

**Keywords:** *Pelargonium* sp., bioactive compounds, sustainable approach, scented plants

## Abstract

This review article aims to present an overview regarding the volatile compounds in different scented species of *Pelargonium* and their biological activities, immunomodulatory activity, cytotoxic activity, high larvicidal activity and ethnopharmacological uses. Although the *Pelargonium* genus includes many species, we focused only on the scented ones, with the potential to be used in different domains. *Pelargonium* essential oil showed great properties as antioxidant activity, antibacterial activity (against *K. pneumonie*, *S. aureus* or *E. coli* strains) and antifungal activity (against many fungi including *Candida* sp.), the responsible compounds for these properties being tannins, flavones, flavonols, flavonoids, phenolic acids and coumarins. Due to the existence of bioactive constituents in the chemical composition of fresh leaves, roots, or flowers of *Pelargonium* sp. (such as monoterpenoid compounds–citronellol, geraniol, linalool, and flavonoids–myricetin, quercetin and kaempferol), this species is still valuable, the bio-compounds representing the base of innovative substitutes in food processing industry, nutraceuticals, or preventive human or veterinary medicine (substitute of antibiotics). Highlighting the volatile chemical composition and properties of this scented plant aims to rediscover it and to emphasize the vast spectrum of health-promoting constituents for a sustainable approach. Future research directions should point to the application of plant biotechnology with a significant role in conservation strategy and to stimulate commercial interest.

## 1. Introduction

*Pelargonium* is a genus comprising approximately 230 perennial plant species [1]. This genus belongs to the family *Geraniaceae* and originally comes from the Cape area in South Africa. Starting from the 18th century, *Pelargonium* has been cultivated in Europe. The name is derived from the Greek word “pelargos” meaning stork and relates to the shape of the geranium flower, resembling a stork’s beak [2]. The *Pelargonium* genus can be categorized into three groups: plants with green or evergreen leaves (*P. graveolens*, *P. quercifolium*, *P. tomentosum*); plants with multi-colored leaves (*P. graveolens* “*Variegatum*”); and plants with flowers and fruits (*P. grandiflorum*-hybrid, *P. peltatum*) [3]. There are many cultivars of this genus, which were derived from approximately 20 species. These cultivars are known to belong to one of six horticultural groups: Angel, Ivy-leaved, Regal, Scented-leaved, Unique, and Zonal [1].

Gunes and Kahraman [4] presented the *Pelargonium graveolens* as being an ornamental species and, at the same time, a plant with edible flowers [4].

Plaschil et al. [5] reviewed the genetic characterization of 15 *Pelargonium* genotypes, resulting in the determination of their ploidy levels. Thus, the species with the highest ploidy levels are *Pelargonium capitatum* (2n = 66), *Pelargonium graveolens*, *Pelargonium vitifolium*, and *Pelargonium radens* (2n = 88). These species could be a fusion of auto allopolyploids [5]. Saraswathi et al. [6] reviewed the phytopharmacological importance of the most important species of *Pelargonium*: *P. graveolens*, *P. reniforme*, *P. sidoides*, and *P. radula*. The genus *Pelargonium* is recognized for its medicinal benefits, and rich sources of monoterpenes, tannins, phenolic acids, cinnamic acids, flavones, flavonoids, coumarins, and flavonol derivatives [6].

Van Wyk [7] presented the importance of some African medicinal plants. *Pelargonium cv. Rosé* leaves have the main use as fragrance, and *Pelargonium sidoides* roots are used in phytomedicine (bronchitis and immune stimulant) and traditional medicine (general tonic, dysentery) [7]. Additionally, Brendler and van Wyk [8] reviewed the historical, commercial, and scientific perspectives of *Pelargonium* species, including antibacterial, antifungal, and immunomodulatory properties [8].

The present review paper aims to present the identified components in different scented species of the *Pelargonium* genus, as well as their potential biological activities, as revealed by scientific papers published in the last decade.

## 2. Results and Discussions

### 2.1. Chemical Composition of Plants from Pelargonium Genus

The rose geranium’s chemical composition is influenced by various environmental elements, including climate, temperature fluctuations, sunlight duration, rainfall levels, phenological stages, harvesting periods and techniques, weed presence, and cultural practices. Pedo-climatic factors influence the quality of the essential oil (EO), in addition to the plant selection and distillation process [9].

Boukhris et al. [10] reported the chemical composition of geranium oil from *P. graveolens* during various phenological stages. In a separate study, Abaas et al. [11] explored the differences in essential oil composition at vegetative and flowering stages, whereas Mahboubi and Valian [12] reviewed the composition and potential applications of nine essential oils obtained from *P. graveolens*. Three types of geranium essential oil were classified by Couic-Marinier and Laurain-Mattar [9]. The three types are: the Chinese variant, which contains a high amount of citronellol (30–40%); the African variant, hailing from Algeria, Morocco, and Egypt, featuring 10-epi-γ-eudesmol (4–5%); and the Bourbon variant, originating from Reunion Island or Madagascar, consisting of a significant amount of guaia-6,9-diene (5–7%), geraniol (15–18%), and linalool (0.5 to 8%) [9].

Eiasu et al. [13] conducted a study on the physio-morphological response of *Pelargonium* plants to irrigation frequency. The results indicated that a high irrigation frequency led to an increase in the favorable ratio of citronellol and geraniol. Furthermore, modifications in essential oil distribution were observed in both glandular and non-glandular trichomes, which resulted in improved functions of plant tissues in the aerial parts (stems, leaves, and floral organs) [13]. Additionally, Lis-Balchin et al. [14] presented the chemical composition and antimicrobial properties of eight distinct *Pelargonium* varieties, while Mehrparvar et al. [15] reviewed the main components present in *P. roseum* Willd that contribute to its antifungal activity.

The composition of primary constituents detected in the essential oil from *Pelargonium* sp. may be impacted by different drying approaches. As a result, the research by Akçura et al. [16] investigated the impact of such methods. It revealed that the shade-drying strategy resulted in the highest concentration of linalool, citronellol, and geraniol, which recorded 7.42 ± 0.44%, 39.87 ± 0.23%, and 17.09 ± 0.12% correspondingly [16].

The featured composition varies depending on various factors, such as the value of the variety, different phenological stages, and seasonal variations. Several studies assessed species belonging to the *Pelargonium* genus during the analyzed period. Table 1 summarizes their primary findings, while the subsequent paragraphs detail the relevant studies.

The primary bioactive components of *Pelargonium* leaves comprise monoterpenoid compounds, including natural acyclic monoterpenoid citronellol (C_10_H_20_O), geraniol (C_10_H_18_O, a monoterpenoid and an alcohol), and linalool (C_10_H_18_O, a monoterpenoid and a tertiary alcohol) [10,14,17]. Additionally, root material contains flavonoids such as myricetin, quercetin, and kaempferol [18].

Another study demonstrated the biosynthesis of citronellol, the primary compound identified in *Pelargonium graveolens*. Banthorpe et al. [19] established that citronellol results from geraniol. This conversion can be achieved by employing a crude enzyme preparation (geraniol reductase) with the ability to reduce the double bond [19].

The chemical composition of fresh leaves of *Pelargonium* sp. is summarized in Table 1 based on multiple scientific studies regarding major volatile components. Geraniol, citronellol, and linalool are the most commonly occurring components.

**Table 1:** Major volatile compounds in different *Pelargonium* species fresh leaves.

Species	Main Identified Volatile Compounds	References
*P. asperum*	Geraniol and β-citronellol	[20]
*P. capitatum*	Citronellol, citronellyl formate, α-pinene, geraniol, geranyl formate and 6,9-guaiadiene	[21]
	Citronellol, citronellyl formate, geranyl formate, β-caryophyllene, 6,9-guaiadiene	
	α-pinene, geranyl formate, β-caryophyllene, 6,9-guaiadiene	
*P.’Chocolate peppermint’*	Menthone (39.1%), isomenthone (22.2%), α-phellandrene (15%), *ρ*-cymene (4.7%)	[14]
*P. cv. Rose*	Citronellol (23.6%), geraniol (12.5%), citronellyl formate (11.1%), linalool (10%), isomenthone (2.7%)	
*P. graveolens*	Citronellol (17.74%) and geraniol (14.73%)	[17]
	Geraniol, citronellol, citronellyl formate, geranyl formate, linalool, 10-epi-γ-eudesmol	[22]
	Citronellol, geraniol, citronellyl formate, L-linalool, 10-epi-γ-eudesmol and geraniol formate	[10]
	Geraniol (18.6–25.5%), citronellol (24.8–28.7%), citronellyl formate (7.9–10.5%), isomenthol (5.4–8.1%) and linalool (1.4–3.4%)	[23]
	Linalool, *cis*-rose oxide, *trans*-rose oxide, menthone, isomenthone, citronellol, geraniol, citronellyl formate, geranyl formate and 10-epi-γ-eudesmol	[24]
	Citronellol, geranial, geraniol, guainene, germacrene D, iso-menthone, geranyl formate	[25]
	Linalool, iso-menthone, citronellol, geraniol, citronellyl formate, geranyl formate, 10 epi-γ-eudesmol	[26]
	Citronellol (15.64%), geraniol (11.31%), citronellyl formate (10.19%), isolongifolan-7-a-ol (7.84%)	[27]
	Citronellol (39.9–49.19%), geraniol (6.5–14.88%), epi-γ-Eudesmol (7.6–10.49%), isomenthone (3.2–6.0%), citronellyl formate (3.6–4.9%) and linalool (1.3–4.9%)	[28]
	β-Citronellol, geraniol, citronellyl formate, linalool, (+)-isomenthone, σ-selinene	[29]
	Citronellol and geraniol	[30]
	Citronellol (32%), geraniol (15%), linalool (6%), isomenthone (6%), geranyl formate (2.5%), tiglate (2%), citronellyl formate (6%), guaia-6,9-diene, and 10-epi-γ eudesmol (5%)	[31]
	Linalool, menthone, geraniol, isomenthone, citronellyl formate, geranyl formate, *cis*-rose oxide, *trans*-rose oxide	[32]
	Citronellol, geraniol, citronellyl formate, iso-menthone, linalool, E-caryophyllene	[33]
	Citronellol (17.74%), geraniol (14.73%), 10-epi-γ-eudesmol (9.52%), citronellyl formate (5.96%), geraniol formate (3.82%), menthone (2.48%), and isomenthone (2.11%)	[34]
	Citronellol, linalool, geraniol, citronellyl formate, geranyl formate, geranyl acetate, limonene, trans-caryophyllene	[12]
	Geraniol, citronellol, β-linalool, γ-eudesmol, citronellyl formate, isomenthone, geranyl tiglate, germacrene-D, geranyl formate	[35]
	Isomenthone (41%), geraniol (19.1%), linalool (12.8%), citronellol (11.6%), citronellyl formate (11.3%)	[14]
	*p*-menthan-3-ol (13.31%), citronellol (27.41%), and geraniol (43.58%)	[36]
	Citronellol (19.22%), geraniol (14.03%) and citronellyl formate (10.02%)	[37]
	Citronellol, menthan-2-one, citronellyl formate, 10-epi-γ-eudesmol, rose oxide B, citronellyl propanoate and citronellyl butanoate	[38]
*P. graveolens cv. Bourbon*	Citronellol and geraniol	[39]
*P. graveolens L’Her*	Linallol, citronellol, geraniol	[40]
	Monoterpenic primary alcohols–citronellol and geraniol	[41]
*P. radens × P. capitatum*	Citronellol, citronellyl formate, β-caryophyllene, germacrene D	[21]
*P. roseum R. Br.*	β-citronellol, citronellyl formate, geraniol, iso-menthone, linalool	[40]
*P. roseum* Willd.	Citronellol (34.22%), geraniol (11.67%), linalool (8.7%)	[15]
*P. x hybridum* cv. ‘*Atomic snowflake*’	Citronellic acid (37%), citronellol (14.8%) + many sesquiterpenes	[14]
*P. x hybridum* cv. ‘*Mabel Grey*’	Citronellal (49.9%), citronellol (37.4%), geraniol (4.1%)	
*P. x hybridum* cv. ‘*Sweet Mimosa*’	Isomenthone (35.7%), β-pinene (15.9%), α-phellandrene (5.9%)	
*P. x hybridum* cv. ‘*Royal Oak*’	Linalool (23%), α-terpinene (7.1%), menthol (3.5%), ρ-cymene-8-ol (2.3%)	
*P. x hybridum* cv. ‘*Clorinda*’	β-pinene 20.1%, α-phellandrene 7.3%, ρ-cymene 5.1%, limonene 4.3%, fenchone 4%	

**Figure 1:**
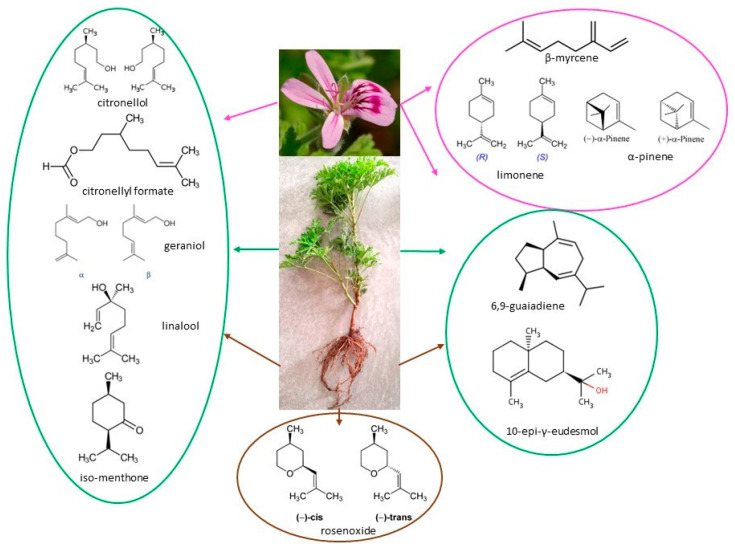
Primary volatile compounds of *Pelargonium* plant species.

Furthermore, Figure 1 presents the primary volatile components of *Pelargonium* plant species, according to data from the literature.

Of the 120 phytoconstituents found in the plant, including sesquiterpenes, monoterpenes, and other important compounds, there are three key components that contribute to its scent: geraniol, citronellol, linalool, and their esters (Table 2). These compounds represent approximately 60% of the total essential oil [42].

The chemical components present in *P. graveolens* can be classified into several categories, including aliphatic hydrocarbons, aromatic hydrocarbons, terpene hydrocarbons, sesquiterpene hydrocarbons, aliphatic alcohols, terpene alcohols, aromatic alcohols, sesquiterpene alcohols, aliphatic esters, aromatic esters, terpene esters, aliphatic ketones, terpene ketones, sesquiterpene ketones, aliphatic aldehydes, terpene oxides, sesquiterpene oxides, aliphatic acids, terpene acids, and miscellaneous compounds [42]. Additionally, Blerot et al. [43] conducted a comprehensive analysis of *Pelargonium* essential oil, outlining the fatty acid derivatives, cyclic monoterpenes and derivatives, acyclic monoterpenes, and derivatives, sesquiterpenes and derivatives, and phenylpropanoid derivatives. The chemical compounds found in *Pelargonium* not only contribute to the flavor and aroma of plants, but also support respiratory systems when used in products. These compounds are responsible for many antibacterial, antiseptic, and antiviral properties.

*Pelargonium* chemical composition is influenced by the variation in environmental factors. The most common factors are related to the climate, such as high and low temperature, the sunny period, rainfalls, different phenological stages, harvesting period or harvested parts, the presence of weeds or cultural. Moreover, these pedoclimatic factors impact the quality of the *P. graveolens* essential oil. Thus, Table 3 illustrates a comparison of essential oil contents from various *Pelargonium* species, ranging from 0.11% to 4.60%.

Taking into consideration all the research articles mentioned in Table 4, the main components were determined using GC-MS, GC-FID, and HPLC.

### 2.2. Biological Activities of Plants from Pelargonium Genus

According to scientific research, *Pelargonium* sp. exhibited antibacterial, antifungal, antitubercular, anticancer, antioxidant, anthelmintic, insecticidal activities, as well as immunomodulatory and cytoprotective properties. All these properties are due to the quality of essential oil. The phenols, polyphenols, tannins, terpenes, ketones, aldehydes, and alcohols are responsible for other potential application [9,42,60,117,121]. Moyo and Van Staden [122] reviewed the medicinal properties and conservation of *Pelargonium sidoides*. In vitro studies revealed that *Pelargonium sidoides* extract has antiviral activity (coronavirus, influenza A viruses), antibacterial activity (*Staphylococcus aureus*, *Escherichia coli* ATCC 11775), antimycobacterial activity (*Mycobacterium smegmatis*), antifungal activity (*Candida albicans*), antiparasitic activity (*Leishmania donovani*) and immunomodulatory activity (*Listeria monocytogenes*, *Leishmania donovani*). Additionally, these studies demonstrated anticoagulant activity, central nervous system activity, and lipopolysaccharide-induced sickness behavior. Thus, *Pelargonium sidoides* extract is utilized in traditional medicine to treat dysentery, diarrhea, common cold, and respiratory infections, bronchitis, tuberculosis, acute rhinosinusitis, and asthma [122].

#### 2.2.1. Antioxidant Properties

Mishra et al. [123] reported on the evaluation of total antioxidant activity assessed by ferric reducing antioxidant power (FRAP) and potassium ferric cyanide (PFC) assays. Regarding non-enzymatic antioxidants, younger leaves of *P. graveolens* showed greater flavonoid accumulation compared to mature leaves and exhibited the strongest reducing power activity. Of the four plants studied (*Moringa oleifera*, *Pelargonium graveolens*, *Tagetes patula* and *Calotropis gigantea*), mature leaves of *P. graveolens* were found to have the second highest phenolic content [123].

In their study, Chrysargyris et al. [124] investigated the correlation between Cu uptake from *Pelargonium graveolens* roots and leaves and its antioxidant activity. Using the antioxidant assays of radical scavenging activity—2,2-diphenyl-1-picrylhydrazyl (DPPH), FRAP or radical scavenging assay—2,20-azino-bis(3-thylbenzothiazoline-6-sulphonic acid (ABTS), Chrysargyris et al. [124] analyzed the effects of copper toxicity on plant growth, plant copper distribution and oxidative stress indicators. The results of the study showed that rose geranium has the ability to accumulate heavy metals in the roots even at copper concentrations of up to 100 μM, leading to an increase in both phenolic content and antioxidant activity [124].

Negro et al. [57] evaluated the antioxidant activity using of DPPH, FRAP, and SASA (Superoxide Anion Scavenging Activity) assays during three different stages of development (mature bud, full bloom, and senescing) in *P. odoratissimum* flowers. Although the lowest phenolic content was observed during the full bloom stage, these flowers exhibited the highest antioxidant capacity. Additionally, the levels of total phenolic compounds were found to be significant. The levels of flavonoids in the flowers of *P.* ‘*Endsleigh*’ were found to be identical to those in both flowers and stems of *P. graveolens* [57].

Cavar and Maksimovic [107] reported on the antioxidant activity of *P. graveolens*. The DPPH assay indicated IC_50_ values of 63.70 mg/mL in leaves and 64.88 mg/mL in stems for essential oils, and 0.19 mg/mL in stems and 0.39 mg/mL in leaves for hydrosol. Analysis of the results led to the conclusion that the EO obtained from the stems had a greater antioxidant activity than that obtained from *Pelargonium* leaves [107].

In addition to *P. graveolens*, other *Pelargonium* species revealed antioxidant activity. Latte and Kolodziej [125] assessed the antioxidant properties of the key components of *P. reniforme* (tannins and flavonoids) through DPPH radical determination and compared them with ascorbic acid, which was used as a positive control. All tested tannin compounds, including corilagin (IC_50_ = 2.7 μM), brevifolincarboxylic acid (IC_50_ = 4.6 μM), phyllanthussin C (IC_50_ = 5.8 μM), methyl gallate (IC_50_ = 6.9 μM), and glucogallin (IC_50_ = 9.9 μM), showed higher inhibitory activity compared to ascorbic acid (IC_50_ = 40.9 μM), excluding gallic acid (IC_50_ = 32.9 μM). Similarly, regarding flavonoids, all components showed higher inhibitory values (with IC_50_ ranging from 2.6 μM for orientin 2″-gallate to 23.2 μM for isoorientin) than ascorbic acid (IC_50_ = 40.9 μM). Therefore, this research demonstrates that *P. reniforme* has antioxidant activity and can potentially be used in the treatment of liver disease [125].

Krishnaiah et al. [126] reviewed the antioxidant properties of various medicinal plant species, including *P. endlicherianum* which is known for its numerous biological activities. In particular, the extract of *P. endlicherianum* exhibited a higher antioxidant activity (IC_50_ = 7.43 ± 0.47 μg/mL) compared to the synthetic antioxidant butylated hydroxytoluene (BTH) (IC_50_ = 18.0 ± 0.4 μg/mL) [126]. Meyers et al. [127] reported that *Pelargonium* species contain significant quantities of phenolic compounds, including hydrolysable tannins and flavonoids, that have antioxidant properties. Among the hundreds of *Pelargonium* subspecies, *P. sidoides* and *P. reniforme* are identified as containing methyl ester and gallic acid in their chemical composition. These two compounds have been found to enhance the immune response [127].

The reviewed articles presented in Table 5 show that various *Pelargonium* sp. plants have antioxidant activity attributed to responsible compounds such as phenols, flavonoids, or tannins. Ascorbic acid was frequently used as a control and in almost all *Pelargonium graveolens* samples the antioxidant capacity exceeded the positive control.

#### 2.2.2. Antimicrobial Activity

The volatile oil of *P. graveolens* was analyzed for chemical composition and assessed for its anti-*Helicobacter* activity using GG-MS. Ninety-two chemical compounds were identified in the oil sample. Among them, citronellol, geraniol, citronellyl-formate, and isolongifolan-7-a-ol were found to be the predominant components, representing 15.64%, 11.31%, 10.19%, and 7.84% of the total, respectively. The EO showed good activity against *H. pylori* with a minimum inhibitory concentration (MIC) of 15.63 mg/mL. Combining the volatile oil with clarithromycin (CLR) resulted in a significant synergistic effect, with a fractional inhibitory concentration index (FICI) of 0.38 mg/mL. The in vitro interaction between *P. graveolens* oil and CLR augmented the antimicrobial activity of the latter, indicating the need for further studies to determine formulations for potential antimicrobial uses [27].

Choi et al. [83] conducted a study on the antimicrobial activity of *P. graveolens* in combination with antibiotics against *S. pneumonia*. The study employed three antibiotics, erythromycin, norfloxacin, and oxacillin, combined with three main compounds identified in *P. graveolens*, citronellol, geraniol, and linalool. The combination of norfloxacin and citronellol demonstrated the strongest synergistic effect with a FICI of 0.16 against four strains of *S. pneumonia* (0.38, 0.31, 0.16 and 0.28, respectively).

Gâlea and Hâncu [137] demonstrated the antiseptic properties of *P. roseum* extract by studying its antibacterial and antifungal effects. They tested the antimicrobial activity on three Gram-negative bacteria, two Gram-positive bacteria, and a fungus, with *P. graveolens* EO exhibiting varying degrees of sensitivity for each bacterial strain. The growth of *C. albicans* was inhibited by 100% in less than 48 h [137].

##### Antibacterial Activity

In most cases, *P. graveolens* EO was obtained through the process of hydro-distillation. Using the MIC and MBC (minimum bactericidal concentrations) assays, *P. graveolens* EO demonstrated an average MIC value of 1%. Furthermore, the average MIC for Gram-positive bacteria was approximately 0.3%, whereas Gram-negative bacteria had an average MIC of 1.5%. *P. aeruginosa* strains were found to be more resistant, with 2% MIC. Concerning Gram-positive cocci, each tested strain demonstrated a low MIC percentage (<0.85%), apart from *S. saprophyticus*, which had a MIC of 1%. The essential oil of *P. graveolens* showed an MBC average of 0.4% against Gram-positive bacteria and more than 4% against Gram-negative bacteria, except for *A. baumannii* strains, for which it was nearly 2%. Citronellol and geraniol were identified as the active compounds responsible for these MIC and MBC values [37].

Extracts of *P. reniforme* and *P. sidoides* have been prepared using ethanol and acetone. They have demonstrated activity at 5 × 10^3^ mg/L against *H. influenzae*, *M. catarrhalis* and *S. pneumoniae*. However, they are not as effective as streptomycin sulphate, which demonstrated activity at 10.0 mg/L and showed complete inhibition activity against these three bacteria [138].

The essential oil extracted from *P. endlicherianum* had a MIC of 5 g/L against *H. influenzae* and 20 g/L against *Neisseria meningitidis*. *Pelargonium* EO combined with ciprofloxacin or ampicillin demonstrated a synergistic effect on *N. meningitidis* and an additive effect on *H. influenzae*. Additionally, the combination of *Pelargonium* EO and gentamicin had a synergistic effect against both meningitis causative pathogens (FICI 0.5). The combinations were tested on human leukocyte cells to determine their effects. The responsible components, determined by MIC and time–kill assays [139], were phenols.

The reviewed literature shows that *Pelargonium* sp. can have comparable results to antibiotic controls. The combination of *Pelargonium* sp. and antibiotics (such as norfloxacin, ciprofloxacin or cefepime) resulted in a synergistic effect on both Gram-positive bacteria (*S. aureus*, *B. cereus*) and Gram-negative bacteria (*E. coli*). This was attributed to the presence of compounds such as phenols, flavonoids, or tannins. The results are shown in Table 6.

##### Antifungal Activity

In their review, Hamidpour et al. [117] state that *P. graveolens* EO has antioxidant, antibacterial, antifungal, and medicinal properties and has been shown to be effective against Gram-negative bacteria such as *E. coli*, *P. vulgaris* and *E. aerogenes* when compared to controls such as chloramphenicol and amoxicillin. In addition, the review specifies that the essential oil extract is more effective in inhibiting the yeast than the bacteria, discussing the effectiveness of *P. graveolens* EO against *Candida tropicalis* and *Candida albicans* yeasts, as well as *Staphylococcus aureus* bacteria [117]. According to Rosato et al. [144], *P. graveolens* EO was the most effective oil among the tested ones. With a minimum inhibitory concentration of a single sample (MIC A) ranging from 0.18 to 0.70 mg/mL, and a minimum inhibitory concentration of a single sample of the most effective combination (MIC B) ranging from 0.04 to 0.28 mg/mL, alongside with FICI values ranging from 0.13 to 0.40 mg/mL, *P. graveolens* oil demonstrated superior efficacy against five strains of *Candida* species (*C. albicans* ATCC 14053, *C. albicans* NRRL y-869, *C. albicans* ATCC 10231, *C. albicans* NRRL y-22077 and *C. guilliermondii* NRRL y-324). Additionally, *P. graveolens* oil demonstrated a strong degree of synergism with amphotericin B [144].

*Pelargonium asperum* EO, together with two other essential oils, has been used at a high concentration of 30% for anti-infective purposes in cases of bacterial, viral, or parasitic dermatitis. The main aim in treating fungal infections was to rapidly eliminate pruritus within 1–2 days using the essential oil’s potent anti-inflammatory and antihistamine properties. *P. asperum* EO has been shown to be effective in stopping progression and initiating regression of various types of allergic conditions such as eczema, psoriasis and dyshidrosis, while also repairing the skin barrier [53].

Table 7 illustrates that all *Pelargonium* species inhibited the growth of several fungal agents through citronellol and geraniol compounds. These results had been obtained via MIC, MFC, ADM and other types of assays.

#### 2.2.3. Other Potential Applications

In their study, Brendler and Van Wyk [8] reviewed the medicinal uses of *Pelargonium* species. Therefore, *Pelargonium* species are acknowledged to aid in the treatment of diarrhea and dysentery (*Pelargonium antidysentericum*), amenorrhea, anemias, and weaknesses (*Pelargonium grossularioides*), animal liver diseases, colic, fever, dysenteries, and diarrheas (*Pelargonium reniforme*), human and cattle dysentery, colic, gonorrhea, worms in calves, and intisila-stomach ailments in babies (*Pelargonium sidoides*) [8]. Referring to *Pelargonium sidoides*, Rachel Wynberg presented its commercial use in the treatment of bronchitis and in South Africa as a traditional medicine [147]. Additionally, Wopker et al. [148] discussed the use of *Pelargonium sidoides* root extract as an alternative medicine for bronchitis treatment in children.

Meyers et al. [127] described the various uses of *Pelargonium* in their book, including culinary, craft, cosmetic, medicinal, ethnobotanical, aromatherapy, and gardening applications (Table 8). Additionally, *Pelargonium* species can be used as insect repellents, agents with a preservative role, tobacco substitutes, or in nanotechnology (Table 9).

Swanepoel [149] specified that *Pelargonium* sp. has numerous potential applications and is currently being utilized in food, cosmetic, and pharmaceutical product compositions [149].

Abdel Rahman et al. [150] investigated the potential effects of *Pelargonium graveolens* essential oil on the toxic impacts of profenofos in common carp. Their findings suggest that the oil could be used as a dietary supplement in aquaculture [150]. The article regarding the effect of *Pelargonium sidoides* extract on growth of crayfish (*Astacus leptodactylus*) also falls in the same field. After 105 days of diets containing *P. sidoides* extract (0, 0.5, 1 and 2 mL × 100 g^−1^), there was an increase in the parameters of weight gain, survival rate, Food Conversion Ratio and Protein Efficiency Ratio. Additionally, the advantages of this experimental diet were observed in the increase in moisture, protein content, as well as the decrease in lipid content [151]. Can et al. [6] proposed that *Pelargonium graveolens* EO exhibited anesthetic properties for two fish species, *Sciaenochromis fryeri* and *Labidochromis caeruleus*, with an optimal concentration of 75 μL × L^−1^. These findings suggest potential use of the EO as an agent for anesthesia and sedation in aquaculture [6].

Naveenkumar et al. [152] identified a method of obtaining an eco-friendly biofungicide used in the treatment of rice seed diseases. The researchers utilized three plant oils—*C. citratus*, *C. martini*, and *P. graveolens*—to create a highly effective emulsifiable concentrate (EC) against *C. lunata*, *F. moniliforme*, *B. oryzae*, and *S. oryzae*. The results indicated that these three oils possess the capacity to suppress mycelial growth of rice seed pathogens. The formula containing 30EC *P. graveolens* essential oil was found to be effective against *C. lunata*, *F. moniliforme*, *B. oryzae*, and *S. oryzae*, inhibiting their growth by 89.8%, 90.7%, 86.6%, and 94.1%, respectively [152].

Lozano-Navarro et al. [153] presented a method for viscosity modification of Mexican superheavy crude oil using an aqueous extract of *Pelargonium hortorum*, a common geranium species. The extract showed efficient dispersion of asphaltenes.

Upadhyaya et al. [154] studied a novel agrotechnology for producing high-quality planting material of *Pelargonium graveolens*. They prepared stem cuttings and planted them below three trees (*Putranjiva roxburghii*, *Bischofia javanica*, *Ficus religiosa*), with necessary irrigation. The raising of cutting in root trainer placed under *Putranjiva roxburghii* showed good results regarding plant height, leaves per plant, and survival rate [154].

Loto et al. [155] studied the electrochemical effects of *Pelargonium* oil concentrates on the corrosion of 1018 carbon steel (high-manganese carbon alloy) in an anionic solution. This study investigated the corrosion inhibition in media containing H_2_SO_4_ 0.5 M and HCl 0.5 M. The electrochemical polarization assay demonstrated that *Pelargonium* oil was highly effective, inhibiting corrosion by 91.56% at a high concentration in H_2_SO_4_, and by 87.32% at 2.5% concentration. ATR-FTIR spectroscopy (Attenuated Total Reflection with Fourier Transform Infrared Spectroscopy) determined an increase in the transmittance of reactive groups in *Pelargonium* concentrates after corrosion. In addition, the inhibition mechanism of *Pelargonium* was revealed by ATR-FTIR spectroscopy. X-ray diffractometry detected corrosive precipitate on the steel, but without concentrate addition [155].

Numerous scientific articles have reported studies on the antioxidant, antibacterial and antifungal properties of *Pelargonium* species. In addition, it was found that rose geranium essential oil (RGEO) possessed anti-inflammatory effects. The application of RGEO at a dose of 200 mL/kg resulted in a reduction in edema by 73%, whereas a dose of 400 mL/kg produced an 88% decrease in edema. These effects were compared to those of the positive control, diclofenac (40 mg/kg), which produced an 85% inhibition of inflammation [66].

Anheyer et al. [156] reviewed *Pelargonium sidoides* as a treatment option for symptoms of respiratory tract infections (RTIs) compared to placebo. The results indicate that *P. sidoides* may be a viable option for treating RTIs in children. Further meta-analyses demonstrate moderate efficacy and safety of the use of *P. sidoides* [156].

*Pelargonium asperum* oil exhibited significant effects when administered either cutaneous or intraperitoneally to mice in response to curdlan intradermal injection-induced inflammation. Geranium oil (GO) was administered intraperitoneally, and the results indicate that GO suppressed neutrophil accumulation. The same result was seen in the use of prednisolone. Maruyama et al. [20] observed a sedative effect and a loss of normal movement following the second administration, indicating that GO suppresses the activity of MPO (human myeloperoxidase) in a dose-dependent manner.

The acaricidal properties of *P. graveolens* extract were observed against mites. First identified research study demonstrates the mite-control activity against *Dermatophagoides farina* and *Dermatophagoides pteronyssinus*. The activity of *P. graveolens* EO was compared with that of commercial acaricides, namely benzyl benzoate and N,N-diethyl-m-toluamide (DEET). The findings showed that the major components of *P. graveolens* were more toxic than the commercial acaricide. In the case of *D. farina*, the most toxic compound was geraniol (LD_50_ of 0.26 µg/cm^2^), followed by other *P. graveolens* compounds, and ultimately benzyl benzoate (LD_50_ of 10.03 μg/cm^2^) and DEET (LD_50_ of 37.12 μg/cm^2^). Similarly, in the case of *D. pteronyssinus*, the most toxic compound was geraniol (LD_50_ of 0.28 µg/cm^2^), followed by benzyl benzoate (LD_50_ of 9.58 μg/cm^2^) and DEET (LD_50_ of 18.23 μg/cm^2^) [102]. In a separate study, it was found that *P. graveolens* EO contains compounds that exhibit acaricidal activity against *Tyrophagus putrescentiae*, a type of food mite. Consequently, *P. graveolens* oil was compared to a commercial acaricide, and the results demonstrated that geraniol (LD_50_ of 1.95 μg/cm^3^), nerol (LD_50_ of 2.21 μg/cm^3^) and citral (LD_50_ of 9.65 μg/cm^3^) were more effective than the positive control, benzyl benzoate (LD_50_ of 11.27 μg/cm^3^) [157].

Fillipova et al. [95] developed a technique to produce toothpaste named “SPLAT Medical Herbs” with essential oils from *Pelargonium graveolens*. Gas chromatography analysis confirmed the presence of geraniol, validating the use of this essential oil. Consequently, the resultant toothpaste has anti-inflammatory, hemostatic and cleaning properties [95].

*Pelargonium graveolens* has been studied, revealing its extract waste as a viable natural dye for wool fabrics. The study analyzed variables such as temperature, pH, and extraction time, which had an impact on the flavonoid, condensed tannin, and polyphenol content, as well as the potassium sulfur ratio (K/S), ultimately affecting the color strength. The most effective results were obtained at pH = 11, a temperature of 100 °C, and an extraction period of approximately 65 min. Based on the findings mentioned above, the optimal K/S value was 115.15. Thus, the hydro-distillation of solid waste produced by *P. graveolens* is a viable solution for coloring wool fabrics naturally [158].

Apart from its medicinal use and other various biological properties, *P. graveolens* is a beneficial plant in sustainable urban horticulture. A SWOT Analysis conducted on the *Aloysia citrodora* plant in co-cultivation with *P. graveolens* demonstrated twelve advantages, including consistent and uniform crop management, pest control, and enhancement of the food chain [159].

Mazeed et al. [160] reviewed the primary objectives of geranium cultivation in India, which include supplying the aroma, pharmaceutical, and cosmetic industries, serving as a potential phyto-accumulator of heavy metals or bioremediation agent, employing distilled waste in vermiculture, and stimulating the economy and employment. To ensure high-quality rose-geranium, the main macronutrients, including phosphorus, nitrogen, potassium, and sulphur, as well as micronutrients such as iron, manganese, and zinc, are essential [160].

**Table 9:** Other biological activities of *Pelargonium* plants, presented in the literature.

Plant	Action	Extraction Method	Assay	Results	Responsible Compound	References
*P. graveolens*	Antagonistic activity	DNA extraction	TSA, King’s B BOX-PCR	In *P. graveolens* roots were found *Aerococcus*, *Agrococcus*, *Bhargavaea*, *Dietzia*, *Klebsiella* and *Solibacillus* species. In *P. graveolens* rhizosphere and root samples, were found *Bacillus*, *Paenibacillus* and *Streptomyces* species. The genus *Bacillus* was found in 56.2% of isolates. Thus, 14 *Bacillus* sp. isolates had antagonistic activity against *Colletotrichum acutatum*, being able to produce indolic compounds, siderophores and mineralized organic phosphate.	NA	[161]
	Anti-dermatophyte activity	NA	mycelium growth inhibition method, micro-broth dilution assay, MFC, MIC	Inhibitory effect of mycelium growth. The main compounds of GO, geraniol and citronellol are useful in cell membrane interference of dermatophytes and in level decreasing of ergosterol content of cells.	Geraniol and citronellol	[12]
	Anti-Inflammatory	Ethanolic extract	MTT assay	Potential level of inhibition of prostanoid production.	Flavonoids (rutin, myricetin, and kaempferol)	[162]
	Antitumor (Anticancer) activity	NA	Trypan Blue assay	The *Pelargonium* EO showed anticancer activity: LC_50_ = 62.50/86.5 µg/mL in NB4/HL-60, thus the using in cancer treatments. Another study revealed that *P. graveolens* has antitumor activity against uterine cervical neoplasia.	Citronellol, *trans*-geraniol	[60]
	Cytotoxicity	Aqueous extract	Cell viability assay-MTT assay	PdNPs synthesis using *P. graveolens* as reducing, capping agent confirmed by FTIR analysis and zeta potential measurements MTT assay showed that the synthesized PdNPs obtained using *P. graveolens* extract exhibited a significant dose-dependent cytotoxicity towards K562 cells. It is found that cell viability of K562 cells is significantly reduced to 57% when exposed to PdNPs of 10 μg/mL.	Polyphenols	[163]
			MTS assay; COX inhibitor screening assay	Cytotoxicity for HeLa, MCF-7, and Hep3B tumor cell lines; reduced tumor cells viability	Citronellol	[73]
	Insecticidal activity	Steam distillation	Area preference method	The 3 tested EOs had a repellent effect against *T. castaneum* and *R. dominica.* For both tested insects, at concentrations 0.24 mg/cm^2^ the repellent activity was 100% for the 3 tested EOs. For *R. dominica*, at the lowest concentration, 0.03%, the repellent activity was 50.5% for the geranium stripping oil, 20% for the geranium oil and 10% for the geranium absolute oil. For *T. castaneum*, at the lowest concentration, 0.03%, the repellent activity was 66.7% for geranium stripping oil and geranium absolute, and 60% for the geranium oil	NA	[84]
		Hydro-distillation	NA	*Pelargonium graveolens* EO acts on fungi such as *C. neoformans*, *C. albicans.* The results of experiments showed that *P. graveolens* essential oil exerts strong activity against all clinical isolates of *S. aureus*, including multidrug-resistant strains, MRSA strains and MLS (B)-positive with values MIC from 0.25 to 2.50 μL/mL	10-epi-γ-eudesmol	[99]
			DBM	The *P. graveolens* EO showed the most toxic values against larvae (LC_50_ = 0.75 μg/μL after 24 h, LC _50_ = 0.49 μg/μL after 48 h, and LC_50_ = 0.36 μg/μL after 72 h), stronger than the positive control (matrine) and then the other 12 plant’s essential oils.	β-citronellol, linalool, and geraniol	[51]
		NA	Bioassays	*P. graveolens* EO showed a high treatment in tick reproduction, but not to inhibit hatchability: Geranium 1% = 85.9%; Geranium 5% = 92.6%; Geranium 10% = 97.0% The other EO (*C. martini*, *C. citratus*, *C. atlantica*) have demonstrated 100% efficacy regardless the concentration.	Citronellol	[164]
			Larval immersion test, adulticidal tests, repulsion test	The different concentration of geranium oil does not show larval mortality (for *M. domestica* and *L. cuprina)* considering Diazinon (1%), the positive control. On the other side, for the adulticidal activity, all the treatments showed in 93–100% mortality.	Citronellol and geraniol (*trans*-geraniol)	[34]
	Phytoremediation activity	Hydro-distillation	ICP-OES, TF, BCF, BAF	*P. graveolens* had the capacity to accumulate high concentrations of heavy metals (chromium 6.6–49.1%, cadmium 40.2–78.9%, lead 20.5–67.6% and nickel 19.3–76.4%) contaminated sludge.	NA	[23]
	Treatments for infertility		Sperm Motility Assay, Hormonal Analysis (ELISA test), Histopathological Investigations	GEO prevents male reproductive disorders by increasing antioxidant capacity, regulates steroidogenesis and mitochondrial biogenesis-related genes. GEO protects against testicular tissue damage caused by TiO_2_ NPs.	Citronellol and geraniol	[91]
*P. graveolens cv. Rosé*	Anti-inflammatory activity	Ethanolic extract, water extract, ethyl acetate extract, chloroforms extract.	Albumin denaturation and heat-induced hemolysis	All extracts showed high inhibition of protein denaturation. The highest activity was from the stem chloroform extract, IC_50_ = 0.86 mg/mL (higher than the positive control, diclofenac IC_50_ = 3.77 mg/mL) and the lowest activity was the leaf aqueous extract, IC_50_ = 5.63 mg/mL. For the heat-induced hemolysis, the best results was obtained using the leaf extractions than the stem extractions. The highest result was in the leaves chloroform extract (IC_50_ = 0.21 mg/mL).	Flavonoids	[133]
	Cytotoxic activity	Ethanolic, water, ethyl acetate, chloroforms extracts	WST-1 cell proliferation assay	The leaves chloroform extract presents the most cytotoxic potential activity with IC_50_ = 0.4 mg/mL	Gallic acid, rutin, quercetin, phenolic compounds and flavonoids	
*P. reniforme* and *P. sidoides*	Antitubercular activity	extracted three times with 1 L of acetone, chloroform, and ethanol.	BACTEC radiometric system	The *P. reniforme* acetone, chloroform and ethanol extracts from roots were active at 5 × 10^3^ mg/L. The positive controls, like streptomycin, ethambutol, rifampicin, and isoniazid showed stronger antitubercular activity than those of the extracts.	NA	[138]
*P. roseum*	Cytotoxic activity	hydro-distillation	Larvicidal bioassay and adulticidal bioassay	Comparing with *Juniperus virginiana*, *Pelargonium roseum* and its components showed higher larvicidal activity against population of *An. gambiae*, in laboratory conditions: LC_50_ = 7.13 ppm (24 h); 1.26 ppm (48 h); 0.90 ppm (72 h)	Sabinene, β-myrcene, bornyl acetate terpinen-4-ol	[116]
	Insecticidal activity		Mosquito rearing, larvicidal assay, ovicidal assay, adulticidal bioassay, ANOVA	*P. roseum* showed mosquito larvicidal activity against *Culex pipiens* species having as mode of action stomach poison.	The lethal concentrations: −7.64 μg/mL (β-citronellol) −6.86 μg/mL (geraniol −14.87 μg/mL (linalool))	[118]
*P. sidoides*	Immune-modulatory or antiviral treatment for SARS-CoV-2 infection	Four extracts: Methanolic, ethyl acetate, n-butanol, and water	ADM	*Pelargonium sidoides* showed immune-modulatory and antiviral properties and it inhibits replication of HCov-229E coronavirus.	Anthocyanins, coumarins, gallic acid, flavonoids, tannins, phenols and hydroxycinnamic acid derivatives	[165]

where: ADM—agar dilution method; ANOVA—analysis of variance; BAF—bio-accumulation factor; BCF—bio-concentration factor; CLR—clarithromycin; COX—cyclooxygenase; DBM—the diamondback moth; DIZ—diameter of the inhibition zone; DMSO—dimethyl sulfoxide; DNA—deoxyribonucleic acid; ELISA—enzyme-linked immunosorbent assay; EO—essential oil; EP-SFME—enzymatic pretreatment combined with solvent-free microwave extraction; EtOAcE—ethyl acetate extract; FICI—fractional inhibitory concentration index; HCov-229E—human coronavirus 229E; HeLa—human cervical; Hep3B-liver; GO—geranium oil; HCNPG—a chitosan hydrogel thickened-nano-emulsion containing *P. graveolens* essential oil; HS—conditions of headspace; IFN—interferon; ICP—OES—inductively coupled plasma-optical emission spectrometry; MBC—minimum bactericidal concentration; MCF—7-breast; MeOHE—methanol extract; MFC—minimum fungicidal concentration; MIC—minimum inhibitory concentration; MLS (B)—Macrolide-lincosamide-streptogramin B; MRSA—methicillin-resistant *Staphylococcus aureus*; MTS-3-(4,5-dimethylthiazol-2-yl)-5-(3-carboxymethoxyphenyl)-2-(4-sulfophenyl)-2H-tetrazolium; MTT-[3-(4,5-2-yl)-2,diphenyltetrazoliumbromide]; NB4/HL-60—two human promyelocytic leukemia cell lines; NEG—nano-emulsions containing geranium; PAE—determination of postantibiotic effect; PdNPs—palladium nanoparticles; SEM—scanning electron microscope; SFE—supercritical fluid extraction; TF—translocation factor; TLC—thin-layer chromatography; TNF—tumor necrosis factor; TSA—tryptic soy agar; UV—ultraviolet; WE—water extract.

## 3. Materials and Methods

The selection of the articles included in this review was performed based on well-known databases (Scopus, Web of Science, ScienceDirect), using specific keywords (“*Pelargonium*”, “*Pelargonium graveolens*”, “geranium”, “composition”, “anti*”, -returning results for “antibacterial”, “antifungal”, “antioxidant activity”).

The validation of the articles was performed manually, inserting only relevant articles with significant contributions to the field of research, resulting in fulfilling this review in its final form.

## 4. Conclusions

The scientific literature presents *Pelargonium* sp.’s biological properties as a potential candidate for employment of rose geranium compounds in alternative medicine, ethnobotanical, plant decoration, and diverse horticultural farming practices. In addition, the pharmacological utility of *Pelargonium* sp. implies the need for friendly conservation approaches within its use. In this sense, applications of plant biotechnology can play a significant role in holistic conservation strategy. Exploring and researching the bioactive principles of interest, including proof-of-concept studies, is necessary to stimulate commercial interest. The identified phytochemicals and their derivatives could thus serve as the foundation for innovative substitutes in various fields, such as the food processing industry, nutraceuticals, or preventive medicine (both human and veterinary).

## Figures and Tables

**Table 2:** The odor of different scented *Pelargonium* plant species.

Species	Odor	References
*P. betulinum*	Camphoreous	[43]
*P. blandfordianum*	Scent of almonds, absinthe, and musk	[44]
*P. capitatum*	Rose	[43]
*P. citronellum*	Lemon	
*P x Citronella hibrid*	Citronella	[44]
*P. crispum*	Lemon, lime	[43]
*P. crispum ‘Variegatum’*	Lemon fragrance	[1]
*P. cucullatum*	Spices	[43]
*P. denticulatum*	Lemongrass	
*P. glutinosum*	Balsamic	
*P. graveolens*	Mint	[43]
	Strong, sweet, and spicy smell	[3]
	Rose	[44]
*P. ‘Graveolens’ of gardens*	Pungent scent of rose lemon	[1]
*P. hispidum*	Fruity	[43]
*P. ‘Lemon Fancy’*	A citrus fragrance	[1]
*P. ‘Mabel Grey’*	A very strong lemon scent	
*P. odoratissimum*	Dill smell	[44]
	A scent reminiscent of stored apples	[1]
*P. papilionaceum*	Lemon	[43]
*P. ‘Prince of Orange’*	Orange-scented leaves	[44]
*P. pseudoglutinosum*	Balsamic	[43]
*P. quercifolium*	Spices	
	Strong, spicy, and hot smell, with a medicinal flavor	[3]
*P. radens*	Mint	[43]
*P. ‘Rober’s Lemon Rose’*	Rose-scented with lemon undertones	[1]
*P. ‘Royal Oak’*	Exotic, spicy scent	
*P. scabroide*	Soap	[43]
*P. scabrum*	Apricot	
*P. ‘Sweet Mimosa’*	Sweet-scented leaves and clusters	[1]
*P. tomentosum*	Mint	[43,44]
	Strong, sweet, and menthol smell	[3]
	Peppermint-scented geranium	[1]
*P. vitifolium*	Lemon balm, lemongrass	[43]

**Table 3:** Oil content in plants from *Pelargonium* sp.

Plant	Part of Plant	Origin of Samples	Oil Content (%)	References
*Pelargonium graveolens T_1_*–*T_6_*	fresh biomass	Bharwara Sewage Treatment Plant (STP), Gomti Nagar Lucknow, India	0.28–0.33	[23]
*Pelargonium graveolens MLE1–MLE4*	leaves	Faculty of Science, Taif University, Saudi Arabia	0.11–0.19	[45]
*Pelargonium graveolens L’Herit.* Bourbon type	aerial parts	CSIR-Central Institute of Medicinal and Aromatic Plants (CIMAP), India	0.20	[30]
*Pelargonium graveolens L’Herit.* CIM-Pawan			0.18	
*Pelargonium graveolens L’Herit.* CIM-Bio-G-171			0.22	
*Pelargonium graveolens L’Herit.* CIMAP Accession-21			0.13	
*Pelargonium graveolens S1–S3*	leaves	El Maamoura (Governorate of Nabeul, North East of Tunisia)	0.35–0.38	[46]
*Pelargonium graveolens S1S2*			0.45	
*Pelargonium graveolens S1S3*			0.47	
*Pelargonium graveolens S2S3*			0.49	
*Pelargonium graveolens S1S2S3*			0.43	
*Pelargonium graveolens in SDS*		The mother plant native to Madagascar	0.13	[47]
*Pelargonium graveolens in BCS*			0.12	
*Pelargonium graveolens in CS*			0.13	
*Pelargonium graveolens* (L’Hér)		Egypt	0.31	[48]
*Pelargonium graveolens* NI and NCG		NA	3.1	[49]
*Pelargonium graveolens* NI and MCT			3.48	
*Pelargonium graveolens* LI and NCG			3.8	
*Pelargonium graveolens* LI and MCT			4.6	
*Pelargonium graveolens*	leaf–flowering stage	CSIR-Indian Institute of Integrative Medicine, Jammu, India	0.16–0.18	[50]
	leaf–over-maturization stage		0.09–0.10	
*Pelargonium graveolens*	leaves	Shanxi Agricultural University, China	2.04	[51]
*Pelargonium graveolens* NM-WW		Isfahan University of Technology, Iran	0.12	[52]
*Pelargonium graveolens* NM-MWD		Isfahan University of Technology, Iran	0.14	
*Pelargonium graveolens*	fresh plant	Burhaniye Aromatic Plants Field Station, Balıkesir Metropolitan Municipality Rural, Turkey	1.98	[16]
	aerial parts	Western Himalayan region, India	0.15	[38]

where: BCS—biochar growth media; CS—control growth media; LI—laser irradiation; MCT—mycorrhizal colonized treatment; MLE1 (1:40), MLE2 (1:30), MLE3 (1:20) and MLE4 (1:10) treatments—the supernatant was diluted with distilled water (*v*:*v*) to reach the required concentrations for foliar spray by adding 1 mL of supernatant into 10, 20, 30, and 40 mL of water; MWD—moderate water deficiency; NCG—non-colonized group; NI—non-irradiated; NM—Non-mycorrhizal; S1, S2, S3—inoculation using the three bacterial strains S1—*Pseudomonas rhizophila* S211, S2—*Halomonas desertis* G11, S3—*Oceanobacillus iheyensis* E9; SDS—solid digestate growth media; T1—100% sewage sludge, T2—80% sewage sludge + 20% soil, T3—60% sewage sludge + 40% soil, T4—40% sewage sludge + 60% soil, T5—20% sewage sludge + 80% soil; T6—soil (only soil); WW—well-watered.

**Table 4:** Chemical composition of *Pelargonium* plant species (as presented by original works published in the period under review).

Specie	Part of Plant	Identified Compounds	Method	References
*P. asperum*	EO	Citronellol, geraniol, citronellyl formate, 6,9-guaiadiene, isomenthone, linalool, geranyl formate, *cis* rose-oxide, α-pinene, *trans* rose-oxide, citronellyl butyrate, geranyl butyrate, geranyl tiglate, phenylethyl tiglate, menthone, isomenthol	GC	[53]
*P. capitatum*	Leaves	α-pinene, 10-epi-γ-eudesmol, citronellol, germacrene D, citronellyl formate, guaia-6,9-diene, δ-cadinene, β-caryophyllene epoxyde	GC-MS	[54]
*P. capitatum × P. radens*	Stem cutting	Linalool, citronellol, citronellyl formate, iso-menthone, geraniol, geranyl formate, guaiadiene (6,9)	GC	[13]
*P. cv. Rosé*	AP	α-pinene, linalool, isomenthone, α-terpineol, citronellol, *trans*-geraniol, citronellyl formate, geranyl formate, β-caryophyllene, isoledene, muurolene, guaia-6,9-diene, oleic acid,3-propyl ester, phnylethyltiglate, eudesmol, cubenol, α-cadinol, emulphor, geranyl tiglate	GC-MS	[55]
*P. endlicherianum*	EO	α-pinene, β-pinene, limonene, β-phellandrene, 2-pentyl furan, *p*-simen, nonanal, dimethyl tetradecane, α,*p*-dimetlystyrene, 2-nonly acetate, bicycloelemen, α-copaene, α-kamfolen aldehyde, pentadecane, decanal, α-burbonen, β-burbonen,trans-α-bergamoten, β-yilangen, β-elemen, β-copaene, β-caryophyllen, undecanal, 6,9-guayadien, aromadendrene, mirtenal, γ-elemen, (e)-2-decenal, γ-gurjunen, (z)-β-farnesene, heptadecane, germacrene d, (e)-2-undecanal, δ-cadenine, γ-cadenine, ar-kurkumen, tridecanal, (e)-geranyl acetone, phenyl ethyl isobutyrate, nonadecane, α-calacorene, 1,5-epoxy-salvial(4)14-en, 2-phenylethyl-2-methylbutyrate, 2-phenylethyl-3-methylbutyrate, caryophyllen oxide, pentadecanal, germacrene D-4β-ol, β-caryophyllenx alcohol, heneicosane, hexahydrofarnesyl acetone, spatulenol, 1-tetradecanol, nor-kopaonon, methylhexadecanoate, α-cadinol, cadalene, guaya-6,10(14)-dien-4β-ol, celine-11-en-4α-ol, tricosenes, farnesil acetone, pentacosenes, dodecanoic acid, phytol, tetradecanoic acid, pentadecanoic acid, nonacosan, hexadecanoic acid	GC-MS, GC-FID	[56]
*P. ‘Endsleigh’*	Flowers	Tartaric acid, malic acid, monogalloylhexose, methylated protocatechuic acid hexose, flavanone hexoside, methyl syringate 4-o-β-d-gentiobiose (leptosperin), myricetin 3-o-glucoside, kaempferol 3-o-di-p-coumaroylhexoside, myricetin-3-o-rhamnoside, myricetin-3-o-rhamnoside is. II, proanthocyanidin, quercetin 3-o-pentoside, kaempferol 3-o-galactoside, kaempferol 3-o-glucoside, kaempferol 3-o-pentoside	HPLC/MS	[57]
*P. graveolens*	AP	α-pinene, camphene, citronellol, citronellal, citronellic acid, geraniol, geranial, linalool, cis-linalool oxide, trans-linalool oxid, 6,9-guaiadiene, 3-carene, δ-carene, limonene, *cis*-ocimene, *cis*-β-ocimene, *trans*-β-ocimene, *cis*-sabinene, α-thujone, *trans*-rose oxide, *cis*-rose oxide, *cis*-limonene oxide, camphor, iso-menthone, p-menthone, p-menthene, menthol, isomenthol, isoborneol, naphthalene, cryptone, z-citral, linalyl acetate, isogeraniol, *trans*-anethole, azulene, geranyl-n-butyrate, geranyl formate, geranyl acetate, geranyl propanoate, geranyl hexanoate, geranyl octanoate, geranyl tiglate, citronellyl hexanoate, citronellyl acetate, citronellyl heptanoate, citronellyl-n-butyrate, citronellyl propanoate, citronellyl propionate, citronellyl formate, iso-menthyl acetate, α-cubebene, β-cubebene, isoledene, α-copaene, β-copaene, isolongifolene, junipene, α-bourbonene, β-bourbonene, *trans*-caryophyllene, calarene, aromadendrene, dehydroaromadendrene, α-guaiene, germacrene-d, α-caryophyllene, β-caryophyllene, seychellene, α-humulene, Ω-cadinene, δ-cadinene, ledene, valencene, δ-himachalene, eremophilene, β-bisabolene, elemol, α-calacorene, *trans*-longipinocarveol, cubenol, ledene oxide, humulene oxide, 10-epi-γ-eudesmol, 6-methyl-5-heptene-2-one, myrcene, α-phelleandrene, ρ-cymene, terpinen-4-ol, neoisomenthol, α-terpineol, α-cubabene, nerol, neral, piperitone, α-agarofuran, gerany valerate, γ-muurolene, α-copene, α-ylangene, terpinolene, iso-isopulegol, phenylethyl tiglate, phenylethyl propanoate, alloarmadendrene, 1,8-cineol, α-cadinol, hinesol, cis-3-hexenol, cis-calamenene, trans-a-bergamotene, α-guaiene, γ-terpinene β-elemene, 1-phenylethyl isobutanoate, caryophylla-4(12),8(13)-dien-5-ol, nonacosane, viridiflorol, α-gurjunene	GC-MS	[58,59,60,61,62,63]
			GC-MS, LC-MS, (HPLC-MS/MS)	[64]
			HPLC	[65]
	EO	Geraniol, citronellol, *p*-menthan-3-ol, nerol, citronellyl propionate, linalool, α-pinene, caryophyllene, menthone, rose oxide	GC	[36]
		α-pinene, myrcene, p-cymene, linalool, *cis*-rose oxide, *trans*-rose oxide, isomenthol, α-terpineol, citronellol, neral, geraniol, geranial, citronellyl formate, citronellyl acetate, citronellyl propionate, geranyl formate, geranyl acitate, geranyl propionate, geranyl triglate, β-bourbonene, (e)-caryophyllene, α-humulene, germacrene d, 2-phenyl ethyl tiglate, 10-epi-γ-eudesmol, geranyltiglate, 6,9-guaiadiene	GC-FID	[23,45]
		Citronellol, linalool, linalool oxide, menthone, isomenthone, geraniol, geraniol formate, geranial, citronellyl acetate, citronellyl formate, citronellyl propionate, citronellyl propanoate, citronellyl tiglate, citronellyl butyrate, citronellyl butanoate, cadinene, α-terpineol, *trans*-rose oxide, *cis*-rose oxide, p-cymene, geranyl propanoate, geranyl acetate, geranyl formate, geranyl tiglate, geranyl butanoate, geranyl n-butyrate, nerol, neral, neryl formate, neryl acetate, α-pinene, β-pinene, geranic acid, (e)-cinnamaldehyde, terpinolene, α-terpinene, limonene, eugenol, borneol, coumarin, 1,8-cineole, carvacrol, α-ylangene, α-copaene, cinnamyl acetate, 10-epi-γ-eudesmol, 6,9-guaiadiene, α-salinene, phenylethanol, caryophellene, α-agarofuran, phenylethyl tiglate, decanoic acid, iso-decanoic, β-bourbonene, α-gumulene,germacrene d, α-bisabolol, β-phellandrene, *cis*-β-ocimene, i-menthol, β-cubebene, z-citral, *trans*-caryophyllene, isoledene, 2-amylfuran, alloaromadendrene, bornylene, β-phenylethyl formate, calamenene, camphene, humulene, myrcene, phenylethyl alcohol, sulcatone, tetrahydro geraniol, phenylacetaldehyde, o-tolualdehyde, *trans*-ocimene, neoisopulegol, citronellal, *trans*-menthan-3-one, neoisomenthol, rhodinol, α-cubebene, β-elemene, longifolene, β-caryophellene, β-copaene, spirolepechinene, *trans*-muurola-3,5-diene, *trans*-prenyl limonene, aromadendrene, γ-muurolene, amorphene, germacerene d, β-selinene, viridiflorene, α-muurolene, *trans*-β-guaiene, 11-norbourbonan-1-one, β-phenylethyl tiglate, geranyl 2-methyl butanoate, β-atlantol, 10-di-epi-cubenol, amorph-4-en-7-ol	GC-MS	[47,49,51,66,67,68,69,70,71,72,73,74,75,76,77]
		Citronellol, geraniol, citronellyl formate, isomenthone, linalool, 10-epi-γ-eudesmol, menthone, geranyl formate, β-bourbonene, menthone, *cis*-rose oxide, β-caryophyllene, geranyl tiglate, geranyl butrate, germacrene D, phenylethyle tiglate, geranyl propionate, citronellyl propionate, geranial, α-copaene, neoisomenthol, α-pinen, α-thuyene, α-terpinol, γ-selilene, *trans*-rose oxide, citronellyl butrate, geranyl acetate, γ-cadinene, calemenene, 6,9-guaiadiene, geranial, 10-epi-γ-eudesmol, 2-phenyl ethyl tiglate	GC-MS GC-FID	[6,30,78]
		Citronellol (14.8–17.4%), *trans*-geraniol (2.10–2.60%), isomenthone (1.30–1.60%), linalool (0.60–0.96%), geranyl acetate (1.00%), γ-cadiene (0.04–0.05%), geranyl butyrate (0.70%), geranyl tiglate (0.07–1.00%), gemacrene D (0.07–0.08%), caryophyllene oxide (1.7–1.8%), geraniol (9.2–11.4%)	GC-MS HPLC	[48]
		α-pinene, 2,2,6-trimethyl-6-vinyltetrahydropyran, limonene, *cis*-linalool oxide (furanoid), *trans*-linalool oxide, linalool, *cis*-rose oxide, *trans*-rose oxide, menthone, isomenthone, menthol, α-terpineol, citronellol, neral, geraniol, geranial, citronellyl formate, neryl formate, geranyl formate, α-cubebene, citronellyl acetate, α-copaene, β-bourbonene, β-elemene, β-caryophyllene, *trans*-α-bergamotene, α-guaiene,6,9-guaiadiene, aromadendrene, α-humulene, allo-aromadendrene, *cis*-muurola-4(14),5-diene,γ-muurolene, geranyl propionate, γ-gurjunene, β-selinene, α-muurolene, γ-cadinene, geranyl isobutanoate, δ-cadinene, citronellyl butanoate, furopelargone A, geranyl butanoate, neryl isovalerate, caryophyllene oxide, 5,5,9,10-tetramethyltricyclo [7.3.0.0(1,6)]dodecan-11-one, 2-phenyl ethyl tiglate, geranyl isovalerate, humulene epoxide II, 1,10-di-epi-cubenol, 1-epi-cubenol, cubenol, α-cadinol, *cis*-citronellyl tiglate, geranyl tiglate	GC-MS, HPLC-UV/Vis	[79]
	Flowers	Citronellol (24.3%), geraniol (21.81%), citronellyl formate (9.94%), linalool (6.67%), 10-epi-γ-eudesmol (5.13%) and p-menthan-3-one (5.03%)	GC-FID GC-MS	[80]
		α-pinene, β-myrcene, β-phellandrene, β-ocimene, linalool, linalool oxide, limonene, *trans*-rose oxide, citronellol, citronellal, l-menthone, l-menthol, β-citronellol, geraniol, citronellyl formate, geranyl formate, geranyl propionate, geranyl butyrate, geranyl tiglate, geranyl acetate, citronellyl acetate, copaene, nerol, lavandulyl acetate, β-bourbonene, β-cubebene, caryophyllene, *trans*-caryophyllene, citronellyl propionate, valencene, isoledene, α-humulene, neoalloocimene, aromadendrene, germacrene-d, β-cuvebene, ledene, α-muurolene, eremophilene, α-amorphene, δ-cadinene, epizonaren, α-agarofuran, phenylethyl tiglate, geraniol butyrate, geraniol formate, 10-epi-γ-eudesmol, mintsulfide, α-terpineol, α-gurjunene, bicyclogermacrene, cadina-1,4-diene, δ-selinene, geranyl isobutyrate, viridiflorol, γ-selinene, β-eudesmol, t-cadinol	GC-MS	[81,82,83]
		Total phenolic content (tannic acid—7.7%), flavones (rutin—0.4%)	HPLC	[65]
	Geranium absolute oil	Limonene (0.13%), *cis*-oxide rose (0.96%), *trans*-oxide rose (0.31%), menthone (0.88%), iso menthone (5.35%), linalool (10%), guaiadiene (0.2%), citronellyl formate (1.19%), geranyl formate (2.19%), citronellol (31.85%), geraniol (22.47%), geranyl butyrate (0.49%), epi-γ-eudesmol (3.24%)	GC-MS	[84]
	Geranium oil	Limonene (0.27%), linalool (4.1%), delta-selinene (9.28%), citronellol (29.76%), geraniol (12.53%), citronellyl formate (7.1%), geranyl formate (2.7%), epi-γ-eudesmol (6.25%), rose oxide *trans* (1.27%), menthone (5.28%), β-bourbonene (1.87%), δ-cadinene (2%), geranyl tiglate (1.46%), phenylethyl tiglate (1.16%)		
	Geranium stripping oil	Pentanal (7.6%), linalool (1.34%), decadienal (5.08%), citronellol (18.33%), geraniol (11.08%), geraniol formate (2.47%), 2,6-octadiene (1.42%), butanoic acid (1.31%), naphthalenemethanol (9.25%), longifolene (1.36%), 1h-cyclopropa(a) naphthalene (2.39%), geranyl tiglate (2.36%), cyclohexanone (3.15%), phenylethyl tiglate (2.39%)		
	Leaves	Citronellol, geraniol, linalool, rose-oxide, 10-epi-γ-eudesmol, geraniol, geranyl formate, geranyl tiglate, citronellyl formate, isomenthone	GC	[59]
		α-pinene, β-pinene, sabinene, myrcene, α-phellandrene, β-phellandrene, limonene, (z)-β-ocimene, (e)-β-ocimene, p-cymene, *cis*-3-hexenol, *cis*-rose oxide, *trans*-rose oxide, *cis*-linalool oxide (furanoid), menthone, *trans*-linalool oxide (furanoid), isomenthone, linalool, β-bourbonene, citronellyl formate, β-caryophyllene, citronellyl acetate, nerodinol, neryl formate, α-humulene, geranyl formate, citronellyl propionate, α-terpineol, germacrene-d, citronellol, geranial, geranyl acetate, piperitone, nerol, citronellyl butyrate, geraniol, geranyl-n-propionate, guaia-6,9-diene, geranyl-n-butyrate, geranyl tiglate, citronellyl tiglate, 10-epi-γ-eudesmol, 2-phenylethyl tiglate, tricyclene, β-copaene, t-elemene, t-cadiene	GC-FID	[85,86,87,88,89]
		α-pinene, β-pinene, β-myrcene, phellandrene, α-phellandrene, β-phellandrene, β-ocimene, linalool, *trans*-rose oxide, *cis*-rose oxide, l-menthone, 3-p-menthanol, α-terpineol, β-citronellol, citral, citronellyl formate, citronellyl acetate, citronellyl propionate, citronellyl ester, citronellyl tiglate, citronellyl butyrate, citronellyl valerate, nerol, geraniol, geranial, geraniol formate, geranyl formate, geranyl acetate, geranyl formate, geranyl tiglate, geranyl propionate, geranyl hexanoate, geranyl butyrate, lavandulyl acetate, β-bourbonene, α-cubebene, β-cubebene, *trans*-caryophyllene, azulene, isoledene, α-humulene, aromadendrene, germacrene-d, ledene, α-muurolene, elemol, α-amorphene, δ-cadinene, γ-cadinene, epizonaren, α-agarofuran, neryl acetate, 2-phenylethyl tiglate, β-selinene, δ-selinene, 10-epi-γ-eudesmol, agarospirol, propanoate, mintsulfide, copaene, isomenthone, isopulegol, e,e-α-farnesene, *cis*-verbenol, *cis*-calamenene, α-agarofurane, linalyl acetate, spatulenol, ο-cymene, β-patchoulene, α-asarone, guaia-6,9-diene, propanoic acid, α-gurjunene, δ.3-carene, menthomenthol, 6-octen-1-ol, 2,6-octadien-1-ol, propionic acid, butyric acid, acide-2-butenoic, ledol, cadina-1,4-diene, valencene, α-cadinol, τ-cadinol, *cis*-ocismene, 3-methylpentane, neohexane, (e)-3,7-dimethylocta-2,6-dien-1-yl dodecanoate, α-iso-pentane, isohexane, trichloromethane, cyclopentane, 1-epi-cubenol, cubenol, 6-methyl-5-hepten-2-one, limonene, citronellal, methyl geranate, 2-phenylethyl isobutyrate, β-elemene, alloaromadendrene, β-selinene, bicyclogermacrene, zonarene, spathulenol, viridiflorol, 2-naphthalenemethanol, cyclohexanone, 2-pentanone, 1,3,6-octatriene, p-menthan-3-ol	GC-MS	[24,36,81,90,91,92,93,94,95,96,97,98,99]
		Citronellol, geraniol, geranial, 10-epi-γ-eudesmol, citronellyl formate, linalool, nerol, neral, isomenthone, isomenthol, α-terpineol, β-citral, cyclofenchene, m-mentha-6.8-diene, *cis*-linalool oxide, *trans*-linalool oxide, geranyl butyrate, geranyl formate, geranyl tiglate, geranyl butanoate, τ-muurolol, (Z)-rose oxide, β-borbonene, caryophyllene, 6,9-guaiadiene, α−humulene, γ-muurolene, bicyclogermacrene, δ-amorphene, inalool isovalerate, 2-phenylethyl tiglate, limonene, menthone, aristolene, neryl propanoate, germacrene-D, α-calacorene, torreyol	GC-MS GC-FID	[80,100,101,102]
		Total phenolic content (tannic acid—22%), phenol carboxylic acids (chlorogenic acid—3.6%), anthocyanins (cyanine chloride—1%)	HPLC	[65]
	Leaves and stems	Geraniol, β-citronellol, citronellyl formate, isomenthone, linalool, germacrene and 10-epi-γ-eudesmol	GC-MS	[103,104]
	Root	Iso-menthone, linalool, citronellyl formate, geranyl formate, citronellol and geraniol	GC	[105]
		Linalool, *cis* + *trans* rose oxide, isomenthone, citronellol, geraniol, citronellyl formate, geranyl formate, geranyl tiglate, 6,9-guadiene, 10-epi-γ-eudesmol	GC-FID	[106,107]
	Sprouts and leaves	Oxygenated monoterpenes, oxygenated sesquiterpenes, sesquiterpene hydrocarbons, citronellol, geraniol, citronellyl formate, linalool	GC-MS GC-FID	[6]
	Stems	β-myrcene, β-ocimene, linalool, *trans*-rose oxide, *cis*-rose oxide citronellal, l-menthone, β-citronellol, geraniol, citral, citronellyl formate, citronellyl tiglate, geranyl formate, α-copaene, β-bourbonene, β-cubebene, caryophyllene, citronellyl propionate, valencene, isoledene, α-humulene, aromadendrene, geranyl acetate, germacrene-D, viridiflorene, α-amorphene, δ-cadinene, geranyl isobutyrate, α-agarofuran, geranyl propionate, geranyl isovalerate, δ-selinene, 10-epi-γ-eudesmol, geranyl butanoate, geranyl tiglate, isomenthone, mintsulfide, cetylic acid, camphor, *trans*-verbenol, 2-methoxy-4-vinylphenol, β-gurjunene, β-ylangene, alloaromadendrene, ledene, *cis*-β-guaiene, α-bisabolene, epi-cubebol, 2-phenylethyl tiglate, isoaromadendrene epoxide, *cis*-phytol, α-pinene, guaia-6,9-diene, *trans*-calamenene	GC-MS	[46,50,81]
		Ethanol, limonene, linalool, phenyl ethanol, *cis*-rose oxide, *trans*-rose oxide, citronellol + nerol, geraniol, eugenol, methyl eugenol, heptadecane, farnesol, nonadecene, nonadecane, eicosane, heneicosane, tricosane, pentacosane, heptacosane	GS-FID	[108]
	AP	Citronellol (24.75%), geraniol (13.99%), γ-eudesmol (11.23%), citronellyl formate (8.37%) and iso-menthone (6.82%)	GC-MS	[64]
	The whole herb	Linalool, isomenthone, β-citronellol, geraniol, geranial, (r)-(+)-citronellic acid, (-)-aristolene, geranyl-*n*-propanoate, β-cubebene, υ-moorolene, geranyl isobutyrate, phenyl ethyl tiglate, υ-eudesmol, τ-cadinol, geranyl tiglate, 2,6-octadien-1-ol, 3,7-dimethyl-acetate, neral, citronellyl formate, 10-epi-γ-eudesmol, geranyl formate, 1,8-cineole, limonene		[109,110]
*P. hispidum*	Dried leaves	Menthone, isomenthone, p-cimene, α-pinene, sabinene, myrcene, phellandrene, carene, terpinene and limonene	GC-MS GC-FID	[111]
*P. hortum*	Fresh leaves	α-thujene, α-pinene, camphene, β-pinene, myrcene, α-terpinene,p-cymene, limonene, ɣ-terpinene, α-terpineol, camphor, α-fenchyl acetate, thymol, bornyl acetate, *trans*-β-caryophyllene, germacrene-D, δ-cadinene, and 5 more unknown components	GC-MS	[112]
*P. odoratissimum*	AP	4-methyl-pentanol, (3E)-hexenol, α-pinene, myrcene, p-cymene, limonene, 1,8-cineole, (Z)-β-ocimene, (E)-β-ocimene, γ-terpinene, *cis*-linalool oxide, linalool, *cis*-thujone, *cis*-rose oxide, *trans*-rose oxide, camphor, neo-isopulegol, menthone, citronellal, iso-menthone, iso-menthol, α-terpineol, citronellol, neral, piperitone, *cis*-myrtanol, geraniol, geranial, citronellyl formate, thymol, geranyl formate, α-cubebene, citronellyl acetate, α-copaene, β-bourbonene, phenyl ethyl isobutanoate, (E)-caryophyllene, α-guaiene, 6,9-guaiadiene, *cis*-muurola-3,5-diene, citronellyl propanoate, *cis*-cadina-1(6),4-diene, germacrene-D, geranyl propanoate, ar-curcumene, phenyl ethyl 3-methyl butanoate, viridiflorene, α-muurolene, geranyl isobutanoate, δ-cadinene, *trans*-cadina-1,4-diene, citronellyl butanoate, furopelargone A, geranyl butanoate, spathulenol, caryophyllene oxide, 2-phenyl ethyl tiglate, geranyl isovalerate, 1,10-di-epi-cubenol, 10-epi-γ-eudesmol, 1-epi-cubenol, epi-α-cadinol, β-eudesmol, α-eudesmol, α-cadinol, (E)-citronellyl tiglate, (E)-citronellyl tiglate	GC-MS	[113]
	EO	Linalool, citronellol, geraniol, citronellyl formate, geranyl formate, β-caryophyllene, germacrene-D, geranyl butyrate, geranyl tiglate, isomenthone, menthone, *trans*-rose oxide, 10-epi-γ-eudesmol		[9,77,114]
	Young leaves	α-pinene, benzaldehyde, sabinene, β-pinene, myrcene, ρ-cymene, limonene, 1,8-cineole, (z)-β-ocimene, (e)-β-ocimene, γ-terpinene, fenchone, linalool, undecane, camphor, isomenthone, borneol, α-terpineol, dodecane, carveol, fenchyl acetate, pipritone, tridecane, α-cubebene, α-copaene, β-cubebene, tetradecane, methyl eugenol, β-caryophyllene, α-caryophyllene, germacrene-D, β-selinene, farnesene, γ-cadinene, germacrene b, caryophyllene oxide, citronyllyl tiglate, octadecane, nonadecane, eicosane		[115]
*P. radens*	Leaves	Tricyclene, α-pinene, β-pinene, myrcene, α-phellandrene, p-cymene + β-phellandrene, limonene, *cis-*β-ocimene, *trans*-β-ocimene, linalool, menthone, isomenthone, α-terpineol, citronellol, piperitone, β-copaene, β-bourbonene, guaiadiene-6,9, germacrene D, t-cadiene, nerodinol	GC-FID	[87]
	Dried leaves	Menthone, isomenthone, p-cimene, α-pinene, rose oxides, sesquiterpenes (guajene, patchoulene, ylangene, cariophyllene oxide)	GC-MS GC-FID	[111]
*P. radula*	RM	Phenolic acids, phenylpropanoids, derivatives (gallic acid, gallic acid methyl ester), coumarins, coumarin glycosides/sulfates, flavan-3-ols/proanthocyanidins, miscellaneous	GC-MS	[18]
	AP	Phenolic acids, phenylpropanoids, derivatives (gallic acid, gallic acid methyl ester, gallic acid ethyl ester, shikimic acid 3-o-gallate, protocatechuic acid, glucogallin), coumarins (scopoletin, umckalin,6,8-dihydroxy-5,7-dimethoxycoumarin, fraxetin, fraxetin-7-β-d-glucoside, magnolioside, 6,7-dihydroxycoumarin-8-sulfate), flavonoids (quercetin, dihydrokaempferol 3-*o*-*β*-d-glucoside, taxifolin-3-*o*-*β*-d-glucoside, luteolin 7-o-β-d-glucoside, vitexin, orientin, isovitexin, isoorientin, epigallocatechin-3-*o*-gallate), miscellaneous		
*P. reniforme*	RM	Phenolic acids, phenylpropanoids and derivates (gallic acid, gallic acid methyl ester, p-hydroxybenzoic acid, protocatechuic acid, vanillic acid, caffeic acid, ferulic acid, *p*-coumaric acid, *p*-coumaraldehyde), coumarins, flavonoids (kaempferol-3-*o*-β-d-glucoside, kaempferol-3-*o*-β-d-galactoside, quercetin-3-*o*-β-d-glucoside, myricetin-3-*o*-β-d-glucoside), flavan-3-ols/proanthocyanidins, miscellaneous		
	AP	Phenolic acids, phenylpropanoids and derivates (gallic acid, gallic acid methyl ester, gallic acid ethyl ester, allic acid butyl ester, shikimic acid 3-*o*-gallate, shikimic acid 3,5-di-*o*-gallate, *p*-hydroxyphenylethanol, *p*-hydroxyphenyl acetic acid, *p*-hydroxyphenyl alcohol, *p*-coumaric acid, *p*-coumaroyl-4-*o*-β-d-glucoside, glycerol-1-gallate, glucogallin, (α,β)-3,4-di-*o*-galloylglucopyranoside, salidroside-6-*o*-gallate), coumarins (scopoletin), flavonoids (kaempferol, quercetin)		
*P. roseum*	AP	α-pinene, linalool, *cis*-rose oxide, *trans*-rose oxide, l-menthone, citronellol, geraniol, citronellyl formate, β-bourbonene, *cis*-calamenene		[116,117]
	EO	Citronellol (44.62%), citronellyl formate (14.42%), geraniol (10.73%), linalool (5.39%), menthone (3.04%), isomenthone (0.89%) and limonene (0.35%)		[77]
	Fresh leaves	α-pinene, β-pinene, β-myrcene, α-phellandrene, p-cymene, D-limonene, β-ocimene, γ-terpinene, linalool oxide, linalool, *cis*-rose oxide, *trans*-rose oxide, β-terpineol, menthone, iso-menthone, menthol, terpinene 4-ol, α-terpineol, β-citronellol, geraniol, α-cubabene, citronellyl acetate, β-bourbonene, β-elemene, α-gurjunene, β-caryophyllene, α-guaiene, β-farnesene, γ-gurjunene, germacrene-D, δ-selinene, valencene, bicyclogermacrene, epizonarene, α-muurolene, α-farnesene, γ-cadinene, δ-cadinene, α-agarofuran, β-eudesmol, cadalene, geranyl tiglate		[118]
*P. sidoides*	Leaf petioles	Phenolic acid compounds (gallic acid, protocatechuic acid, 4-hydroxybenzoic acid, vanillic acid, caffeic acid, p-coumaric acid, ferulic acid and salicylic acid)	UPLC-MS/MS	[119]
*P. x hybridum cv. ‘Rosat Bourbon’*	EO	*cis*-3-hexanol, α-pinene, β-pinene, α-phellandrene, α-terpinene, *para*-cymene, β-phellandrene, limonene, terpinolene, menthone, isomenthone, menthol, α-terpineol, bois de rose oxide, myrcene, *cis*-*β*-ocimene, *trans*-*β*-ocimene, linalool oxide I, linalool oxide II, linalool, rose oxide *cis*, rose oxide *trans*, citronellol, piperitone, geraniol, geranial, citronellyl formate, citronellyl acetate, neryl acetate, geranyl acetate, citronellyl propionate, geranyl propionate, geranyl isobutyrate, citronellyl butyrate, geranyl butyrate, geranyl valerate, citronellyl caproate, citronellyl tiglate, geranyl tiglate, geranyl caproate, α-cubebene, α-copaene, β-bourbonene, β-caryophyllene, α-*trans*-bergamotene, guaia-1(5),11-diene, 6,9-guaiadiene, aromadendrene, α-caryophyllene, alloaromadendrene, β-cubebene, germacrene D, β-selinene, viridiflorene, γ-cadinene, calamenene, δ-cadinene, furopelargone, β-caryophyllene oxide, phenethyl propionate, β-phenethyl isobutyrate, phenethyl tiglate	GC-MS	[43]
*P. x hybridum cv. ‘Rosat China’*		*cis*-3-hexanol, α-pinene, β-pinene, α-phellandrene, α-terpinene, *para*-cymene, β-phellandrene, limonene, terpinolene, menthone, isomenthone, menthol, α-terpineol, bois de rose oxide, myrcene, *cis*-β-ocimene, *trans*-β-ocimene, linalool oxide I, linalool oxide II, linalool, rose oxide *cis*, rose oxide *trans*, citronellol, piperitone, geraniol, geranial, citronellyl formate, citronellyl acetate, neryl acetate, geranyl acetate, citronellyl propionate, geranyl propionate, geranyl isobutyrate, citronellyl butyrate, geranyl butyrate, geranyl valerate, citronellyl caproate, citronellyl tiglate, geranyl tiglate, geranyl caproate, α-cubebene, α-copaene, β-bourbonene, β-caryophyllene, α-*trans*-bergamotene, guaia-1(5),11-diene, 6,9-guaiadiene, aromadendrene, α-caryophyllene, alloaromadendrene, β-cubebene, germacrene d, β-selinene, viridiflorene, γ-cadinene, calamenene, δ-cadinene, furopelargone, β-caryophyllene oxide, *epi*-γ-eudesmol, phenethyl propionate, β-phenethyl isobutyrate, phenethyl tiglate		
*P. x hybridum cv. ‘Rosat Egypt’*		*cis*-3-hexanol, α-pinene, β-pinene, α-phellandrene, α-terpinene, *para*-cymene, limonene, terpinolene, menthone, isomenthone, menthol, α-terpineol, bois de rose oxide, myrcene, *cis*-β-ocimene, *trans*-β-ocimene, linalool oxide I, linalool oxide II, linalool, rose oxide *cis*, rose oxide *trans*, citronellol, piperitone, geraniol, geranial, citronellyl formate, citronellyl acetate, geranyl acetate, citronellyl propionate, geranyl propionate, geranyl isobutyrate, citronellyl butyrate, geranyl butyrate, geranyl valerate, citronellyl caproate, citronellyl tiglate, geranyl tiglate, geranyl caproate, α-cubebene, α-copaene, β-bourbonene, β-caryophyllene, α-*trans*-bergamotene, guaia-1(5),11-diene, 6,9-guaiadiene, aromadendrene, α-caryophyllene, alloaromadendrene, β-cubebene, germacrene d, β-selinene, viridiflorene, γ-cadinene, calamenene, δ-cadinene, β-caryophyllene oxide, *epi*-γ-eudesmol, γ-eudesmol, phenethyl propionate, β-phenethyl isobutyrate, phenethyl tiglate		
*P. graveolens*		α-pinene, β-pinene, limonene, cymol, cineol, terpinen, linalool, camphor, borneol, citronellol, geraniol, neral, geranial	GC-FID	[120]

where: AP—aerial parts; EO—essential oil; GC-FID—gas chromatography–flame ionization detector; GC-MS—gas chromatography–mass spectrometry; HPLC—high-performance liquid chromatography; HPLC-MS—high-performance liquid chromatography with mass spectrometry; HPLC-MS/MS—liquid chromatography with tandem mass spectrometry; HPLC-UV/VIS—high-performance liquid chromatography equipped with UV/VIS detector; LC-MS—liquid chromatography–mass spectrometry; RM—root material.

**Table 5:** Antioxidant properties of different extracts obtained from *Pelargonium* plant species.

Species	Plant Part	Responsible Compounds	Extraction Method	Antioxidant Assay	Antioxidant Potential	References
* P. betulinum *	aerial parts	flavonoids, tannins	acetone extraction	DPPH	IC_50_ = 4.13 μg/mL	[128]
* P. citronellum *					IC_50_ = 23.70 μg/mL IC_50_ = 84.01 μg/mL	
* P. cordifolium *					IC_50_ = 5.01 μg/mL	
* P. crispum *					IC_50_ = 4.49 μg/mL	
* P. cucullatum *					IC_50_ = 40.18 μg/mL IC_50_ = 10.91 μg/mL	
*P. graveolens*	leaves	phenolic compounds	methanol extraction	Phenol and flavonoids content assay, Enzyme activity assay (CAT, APX, GPX, SOD activities), MDA and H_2_O_2_ assays	The EO and total phenol and flavonoids contents increased significantly by 12.4% and 16%, respectively, when *P. graveolens* was underwater deficit 75% FC. The AMF inoculation treatment improved the plant enzymatic defence. Also, AMF inoculation treatment showed a lower MDA and H_2_O_2_ accumulation in plant tissue.	[129]
			methanol extraction ethanol extraction aqueous extraction	DPPH	IC_50_ = 12.24 μg/mL (methanol extraction) IC_50_ = 14.6 μg/mL (ethanol extraction) IC_50_ = 39.45 μg/mL (aqueous extraction)	[80]
				ABTS	IC_50_ = 241.83 μg/mL (methanol extraction) IC_50_ = 235.86 μg/mL (ethanol extraction) IC_50_ = 140.57 μg/mL (aqueous extraction)	
			heat reflux extraction	ABTS DPPH FRAP CUPRAC	TEAC ABTS = 223.76 μM TE/g FW TEAC DPPH = 121.26 μM TE/g FW TEAC FRAP = 231.64 μM TE/g FW TEAC CUPRAC = 176.98 μM TE/g FW	[130]
			methanol extraction aqueous extraction	DPPH	IC_50_ = 20.71 μg/mL (methanol extraction) IC_50_ = 16.59 μg/mL (aqueous extraction)	[64]
				ABTS	0.86 mM of Trolox (methanol extraction) 0.93 mM of Trolox (aqueous extraction)	
	flowers	phenolic compounds	methanol extraction ethanol extraction aqueous extraction	DPPH	IC_50_ = 16.03 μg/mL (methanol extraction) IC_50_ = 19.31 μg/mL (ethanol extraction) IC_50_ = 44.24 μg/mL (aqueous extraction)	[80]
				ABTS	IC_50_ = 233.74 μg/mL (methanol extraction) IC_50_ = 227.73 μg/mL (ethanol extraction) IC_50_ = 131.54 μg/mL (aqueous extraction)	
			methanol extraction aqueous extraction	DPPH	IC_50_ = 10.3 μg/mL (methanol extraction) IC_50_ = 12.85 μg/mL (aqueous extraction)	[64]
				ABTS	1.13 mM of Trolox (methanol extraction) 0.98 mM of Trolox (aqueous extraction)	
	aerial parts	phenolic compounds	methanol extraction dichloromethane extraction hexane extraction	DPPH	IC_50_ = 12.96 μg/mL (methanol extraction) IC_50_ = 116.91 μg/mL (dichloromethane extraction) IC_50_ = 37.6 μg/mL (hexane extraction)	[131]
				ABTS	IC_50_ = 10.2 μg/mL (methanol extraction) IC_50_ = 10.46 μg/mL (dichloromethane extraction) IC_50_ = 44.46 μg/mL (hexane extraction)	
				CUPRAC	IC_50_ = 20.29 μg/mL (methanol extraction) IC_50_ = 53.36 μg/mL (dichloromethane extraction) IC_50_ = 89.85 μg/mL (hexane extraction)	
		flavonoids and condensed tannins	decoction	DPPH	136.1 mg TE/g DM	[132]
		flavonoids, tannins	acetone extraction		IC_50_ = 14.49 μg/mL	[128]
		phenolic compounds and flavonoids	hydro-distillation	DPPH	IC_50_ = 138.23 μg/mL (vegetative stages) IC_50_ = 119.49 μg/mL (beginning flowering stage) IC_50_ = 83.26 μg/mL (full flowering stage)	[60]
				FRAP	IC_50_ = 151.21 μg/mL (vegetative stages) IC_50_ = 139.35 μg/mL (beginning flowering stage) IC_50_ = 116.42 μg/mL (full flowering stage)	
				ABTS	IC_50_ = 174.95 μg/mL (vegetative stages) IC_50_ = 153.39 μg/mL (beginning flowering stage) IC_50_ = 132.25 μg/mL (full flowering stage)	
				H_2_O_2_	IC_50_ = 77.35 μg/mL (vegetative stages) IC_50_ = 64.81 μg/mL (beginning flowering stage) IC_50_ = 48.67 μg/mL (full flowering stage)	
		total phenolic content	ethanolic extraction	ABTS	IC_50_ = 17.53 µg/mL	[65]
				FRP	IC_50_ = 74.43 µg/mL	
*P. graveolens cv. Rosé*	leaves	phenolic compounds	ethanolic extraction	DPPH	IC_50_ = 7.88 µg/mL	[133]
				β-carotene bleaching assay	IC_50_ = 78.3 µg/mL	
				FRAP	IC_50_ = 143 µg/mL	
				H_2_O_2_	IC_50_ = 2533 µg/mL	
	stems			DPPH	IC_50_ = 10.00 µg/mL	
				β-carotene bleaching assay	IC_50_ = 533.4 µg/mL	
				FRAP	IC_50_ = 137.2 µg/mL	
				H_2_O_2_	IC_50_ = 3550.00 µg/mL	
* P. glutinosum *	aerial parts	flavonoids, tannins	acetone extraction	DPPH	IC_50_ = 16.41 μg/mL IC_50_ = 29.17 μg/mL	[128]
* P. hermanniifolium *					IC_50_ = 13.50 μg/mL	
* P. hispidum *					IC_50_ = 12.78 μg/mL	
* P. panduriforme *					IC_50_ = 91.58 μg/mL	
* P. papilionaceum *					IC_50_ = 81.24 μg/mL	
* P. pseudoglutinosum *					IC_50_ = 52.38 μg/mL	
*P. purpureum*	dried leaves	phenolic compounds	infusion	FRAP	487 μM Fe^2+^	[134]
* P. quercifolium *	aerial parts	flavonoids, tannins	acetone extraction	DPPH	IC_50_ = 17.15 μg/mL IC_50_ = 61.87 μg/mL	[128]
*P. radula*	leaves	phenols, tannins and flavonols	ultrasonic extraction–water solvent ultrasonic extraction–ethanol solvent decoction–water solvent	DPPH	IC_50_ = 56.7 μg/mL (water solvent) IC_50_ = 70.3 μg/mL (ethanol solvent) IC_50_ = 28.6 μg/mL (decoction)	[135]
				SRP	IC_50_ = 0.69 μg/mL (water solvent) IC_50_ = 0.95 μg/mL (ethanol solvent) IC_50_ = 1.21 μg/mL (decoction)	
				ANT	IC_50_ = 56.8 μg/mL (water solvent) IC_50_ = 81.3 μg/mL (ethanol solvent) IC_50_ = 77.6 μg/mL (decoction)	
	dried leaves			DPPH	IC_50_ = 41.7 μg/mL (water solvent) IC_50_ = 54.3 μg/mL (ethanol solvent) IC_50_ = 38.9 μg/mL (decoction)	
				SRP	IC_50_ = 1.00 μg/mL (water solvent) IC_50_ = 1.12 μg/mL (ethanol solvent) IC_50_ = 1.14 μg/mL (decoction)	
				ANT	IC_50_ = 75.2 μg/mL (water solvent) IC_50_ = 79.9 μg/mL (ethanol solvent) IC_50_ = 12.2 μg/mL (decoction)	
* P. scabrum *	aerial parts	flavonoids, tannins	acetone extraction	DPPH	IC_50_ = 7.15 μg/mL	[135]
* P. sidoides *	NA	total phenolic content, condensend tannins	50% methanol extraction	DPPH	IC_50_ = 6.64 μg/mL	[136]
				BHT	IC_50_ = 2.66 μg/mL	
* P. sublignosum *	aerial parts	flavonoids, tannins	acetone extraction	DPPH	IC_50_ = 17.61 μg/mL	[128]

where: ABTS—radical scavenging assay-2,20-azino-bis(3-thylbenzothiazoline-6-sulfonic acid; AMF—arbuscular mycorrhizal fungi; ANT—antioxidant activity in β-carotene-linoleic acid assay; APX—ascorbate peroxidase; CAT—catalase; CUPRAC—Cupric reducing antioxidant capacity; DM—dry matter; DPPH—radical scavenging activity-2,2-diphenyl-1-picrylhydrazyl; EO—essential oil; FC—field capacity; FRP—ferric reducing power; FRAP—ferric reducing antioxidant power assay; FW—fresh weight; GPX—glutathione peroxidase; IC_50_—half maximal inhibitory concentration; LP—lipid peroxidation assay; MDA—malondialdehyde; NA—not available; SOD—superoxide dismutase; SRP—slope of trend line in reducing power assay; TE—Trolox equivalent; TEAC—Trolox equivalent antioxidant capacity.

**Table 6:** Antibacterial properties of different extracts obtained from *Pelargonium* plant species.

Plant	Extraction Method	Assay	Results	Responsible Compound	References
*P. endlicherianum Fenzl.*	Hydro-distillation	MIC, ADD, MBC, time–kill assay, PAE, SEM	The antibiotics used in combination with the EO can be effective in the treatment against *Klebsiella pneumoniae.* The time–kill assay detected the bactericidal effects of that combination. Considering the obtained results, cefepime and essential oil presented a synergistic effect against *K. pneumoniae.*	β-bourbonene, α-pinene, β-pinene	[56]
*P. graveolens*	Hydro distillation	MIC and MBC	The MICs values ranging from 0.15 to 2.5 μg/mL, showed that essential oils are effective as antimicrobial agents, and the MIC/MBC ratio are very close to 1, confirming their bactericidal activity.	β-citronellol	[10]
		Disk diffusion assay; vapor diffusion assay	Among the Gram-negative bacteria, the EO was more effective against *E. coli* and *E. aerogenes*. Among the Gram-positive bacteria, *S. aureus* ATCC 6538 and *E. faecalis* ATCC 29212 (DIZ 21.17 mm) were the most sensitive strains to the EO.	Citronellol, geraniol and their esters	[59]
		MIC	Good activity against *H. pylori* at a MIC of 15.63 mg/mL. Once combined the volatile oil with CLR, a significant synergistic effect appeared at a FICI of 0.38%.	Citronellol, geraniol, citronellyl formate, isolongifolan-7-a-ol	[27]
		MIC, MBC	The most active was citronellol and the lowest MIC was found against *E. coli* (0.007 ± 0.0003 mg/mL).	Citronellol	[140]
		ADM	*P. graveolens* has the most limited spectrum of activity, comparing with the other studied plants (*T. vulgaris*, *O. vulgare*, *S. aromaticum*, *M. fragrans*, *P. nigrum*).	Phenolic compounds	[141]
		MIC ADM	The effect increased when the *P. graveolens* EO was combined with Norfloxacin. The results showed a synergism between them, against *B. cereus* ATCC 11778, *S. aureus* ATCC 6538 and *S. aureus* ATCC 29213, with FICI of 0%, 50%, 0.37%, 0.38%, respectively.	NA	[41]
	Steam distillation	ADM, MIC, MFC	The Gram-positive bacteria were resistant to EO, with one exception, *S. aureus*, which was the most sensitive. The high antimicrobial activity is associated with the high contents of oxygenated monoterpenes.	Oxygenated monoterpene	[93]
		MIC, MBC	The highest antibacterial activity was obtained for *S. tiphi*: MIC = 7 mg/mL; MBC > 14 mg/mL. The lowest antibacterial activity was obtained for *E. coli*: MIC = 0.870 mg/mL; MBC 0.878 mg/mL.	Hexadecanonic acid	[11]
		MIC, FICI	*P. graveolens* EO exerts strong activity against all clinical isolates of *S. aureus* with MIC values from 0.25 to 2.50 µL/mL. The FICI for *K. pneumoniae* and *P. mirabilis* was 0.375%, while for *S. aureus* FICI was 0.5%. The FICI values for the tested microorganisms were <0.5%, indicating synergy between *P. graveolens* EO and ciprofloxacin.	β-citronellol	[99]
	EP-SFME and HD	MIC, MBC, MFC	The GEOs from the EP-SFME and HD methods had the best antimicrobial effect on *Escherichia coli* with MIC and MBC of 6.25 and 12.5 mg/mL, respectively.	Citronellol, geraniol	[24]
	Methanolic extract	ADD, MIC	Inhibitory effect was exerted by the extracts against urease and tyrosinase, with IC_50_ values of 31.05 ± 3.76 μg/mL and 21.11 ± 0.38 μg/mL, respectively.	Phenolic, flavonoids, flavonols, tannins	[130]
	Aqueous extract	Microdilution method	Inhibition activity against the COX-1 enzyme.	Citronellol	[73]
	Decoction, infusion, heat reflux	ADM	The *P. graveolens* antimicrobial activity was evaluated against four bacteria species (*S. aureus*, *L. monocytogenes*, *E. coli*, *S. enterica*). The highest inhibitory effect of *P. graveolens* was against *L. monocytogenes.*	Phenolic compounds	[131]
*P. reniforme* and *P. sidoides*	Methanolic extracts	PRB, BMD, ADM	*P. sidoides* showed high inhibitory activity (96%) against *Mycobacterium tuberculosis. P. reniforme* was inactive.	Phenols and coumarins	[121]
*P. sidoides*	NA	DDT	*P. sidoides* had significant anti-adhesive activity agains *H. pylori* and it can be a useful choice in avoiding the first steps of a bacterial infection.	Polymeric proanthocyanidins	[142]
*P. zonale*	Ethanol and acetone extracts	MIC	The *P. zonale* leaves were the most effective against *R. pseudosolanacearum*. The MIC of *P. zonale*, started at 6.25 mg/mL. *P. zonale* had similar results/values like controls (KOBE 1.2 SL-Chrysophanol 12 g/L and ENRICH VM—Bronopol 27% *w*/*w*).	NA	[143]

where: ADD—agar disk diffusion; ADM—agar diffusion method; BMD—broth microdilution; DDT—disk diffusion test; EO—essential oil; EP-SFME—enzymatic pretreatment combined with solvent-free microwave extraction; FICI—fractional inhibitory concentration index; GEO—geranium essential oil; IC_50_ the half-inhibition concentration; MBC—minimum bactericidal concentration; MDA—malondialdehyde; MFC—minimum fungicidal concentration; MIC—minimum inhibitory concentration; NA—not available; PAE—Determination of post-antibiotic effect; PRB—primary radiorespirometric bioassay; SEM—scanning electron microscopy.

**Table 7:** Antifungal properties of different extracts obtained from *Pelargonium* plant species.

Plant	Extraction Method	Assay	Results	Responsible Compound	References
*P. graveolens*	NA	Microdilution method and macrodilution method, MIC, MFC	The *Pelargonium* EO showed high antifungal activity for *C. albicans*, *C. fulvum*, *P. macdonaldii*, *T. menthagrophytes* than the bifonazole (used as control)	Citronellol and geraniol	[79]
	HS extraction-stirring time of 10 min., heating temperature of 70 °C, under 500 rpm	MIC	The most active EO was the sample from South Africa, with MIC between 128 and 256 μg/mL. MIC values obtained for HCNPG were lower for the most part of isolates tested, reaching 8 μg/mL for *C. albicans* and *C. glabrata*		[145]
	NA	Crystal violet, total protein, and ATP-bioluminescence assays	Reduction in antibiofilm treated with GO and NEG; reduction in protein on the plates and catheters; GO and NEG showed lower MIC for *C. albicans* and *C. tropicalis*.		[17]
	hydro-distillation	MIC, MFC	*cis*-menthone was the most active against selected fungi (MIC: from 0.07 ± 0.01 to 0.17 ± 0.01 mg/mL); linalool was active against oral *C. albicans*	*cis*-menthone, linalool	[140]
	SFE, hydro-distillation, maceration	Insecticidal tests, antifungal tests (MIC)	*P. graveolens* essential oil obtained by hydro-distillation had the highest acute toxicity; thus, it can be used as botanical pesticides	*trans*-nerolidol, geraniol and citronellol	[146]
*P. odoratissimum*	NA	ADM	*P. odoratissimum* EO showed higher inhibition effect against fungal species growth. *P. odoratissimum* inhibited the growth of 3 fungal agents at 1 μL/mL (*O. yallundae*, *Z. tritici*, *P. teres*) by 100%.	Phenolic compounds	[114]
*P. reniforme* and *P. sidoides*	Ethanol and acetone extracts	PDA, ANOVA and Duncan’s multiple range test	The *P. sidoides* ethanol extract and *P. reniforme* ethanol and acetone extracts showed activity against fungal pathogens at a concentration of 5 × 10^3^ mg/L. Amphotericin B was active at 0.5 mg/L on each fungus.	NA	[138]

where: ADM—agar diffusion method; ANOVA—analysis of variance; ATP—Adenosine Triphosphate; EO—essential oil; GO—geranium oil; HCNPG—hydrogel-thickened nano-emulsion; HS—conditions of headspace MBC—minimum bactericidal concentration; MDA—malondialdehyde; MFC—minimum fungicidal concentration; MIC—minimum inhibitory concentration; NA—not available; NEG—nano-emulsions containing geranium; PDA—pile driving analysis; SFE—supercritical fluid extraction.

**Table 8:** Main *Pelargonium* uses (from Meyers et al., 2006 [127]).

Plant	Product Obtained
**CULINARY USE**	
*P. acetosum*	Salads or cooked into soups and stews
*P. bowkeri*	Salad herb
*P. citronellum*	Lemon liqueur
*P. ‘Ginger’ (syn. P. ‘Torento’)*	Cakes, jellies, beverages, desserts, and sandwiches
*P. graveolens*	Baked goods, gelatin, pudding, candy, frozen dairy desserts, and alcoholic and non-alcoholic beverages
*P. ‘Nutmeg’*	Cakes, pâté, stuffing, potato salad and coffee
*P.* *odoratissimum*	Fruit drinks, syrups, sauces, and desserts
**COSMETIC USE**	
*P. capitatum*	Perfumery
*P. inquinans*	Deodorant
**MEDICINAL, ETHNOBOTANICAL USES AND AROMATHERAPY**	
*P. alchemilloides*	Root infusion in treating diarrhea Leaf juice in treating the eyes Root decoction in treating fever
*P. antidysentericum*	Decoction used in treating diarrhea Leaf tea used in treating nausea, diarrhea and dysentery
*P. bowkeri*	Treatment for colic, diarrhea
*P. botulinum*	Leaf decoction treatment of dermatological conditions, colds, respiratory infections, sinusitis
*P. capitatum*	Leaf infusion use in the treatment of urinary bladder and kidney diseases
*P. cucullatum*	The crushed leaves used in treating insect bites, bruises, boils, wounds The leaf infusion used in treating fever, diarrhea, abdominal pain
*P. graveolens*	The leaf infusion used in treating insomnia, dysentery, diarrhea, vomiting Inhalation used to treat asthma
*P. luridum*	Root infusion used in abdominal pains, dysentery, diarrhea, backache Leaf infusion or powdered root used in fever, abdominal pains
*P. quercifolium*	Remedy for rheumatism, heart disease
*P. reniforme*	The decoction used in dysentery and diarrhea
*P. sidoides*	The decoction used in different parasitic zoonoses

## Data Availability

Data sharing not applicable.
